# Mitochondrial biogenesis in white adipose tissue mediated by JMJD1A-PGC-1 axis limits age-related metabolic disease

**DOI:** 10.1016/j.isci.2024.109398

**Published:** 2024-03-01

**Authors:** Ryo Ito, Shiyu Xie, Myagmar Tumenjargal, Yuto Sugahara, Chaoran Yang, Hiroki Takahashi, Makoto Arai, Shin-Ichi Inoue, Aoi Uchida, Kenji Nakano, Hyunmi Choi, Ge Yang, Yanan Zhao, Rei Yamaguchi, Hitomi Jin, Hina Sagae, Youichiro Wada, Toshiya Tanaka, Hiroshi Kimura, Tatsuhiko Kodama, Hiroyuki Aburatani, Kazuhisa Takeda, Takeshi Inagaki, Timothy F. Osborne, Takeshi Yoneshiro, Yoshihiro Matsumura, Juro Sakai

**Affiliations:** 1 Division of Molecular Physiology and Metabolism, Tohoku University Graduate School of Medicine, Sendai 980-8575, Japan; 2 Division of Metabolic Medicine, Research Center for Advanced Science and Technology, The University of Tokyo, Tokyo 153-8904, Japan; 3 Isotope Science Center, The University of Tokyo, Tokyo 113-0032, Japan; 4 Department of Nuclear Receptor Medicine, Laboratories for Systems Biology and Medicine, Research Center for Advanced Science and Technology, The University of Tokyo, Tokyo 153-8904, Japan; 5 Cell Biology Unit, Institute of Innovative Research, Tokyo Institute of Technology, Yokohama 226-8503, Japan; 6 Genome Science and Medicine Division, Research Center for Advanced Science and Technology, The University of Tokyo, Tokyo 153-8904, Japan; 7 Graduate School of Nursing, Miyagi University, Miyagi 981-3298, Japan; 8 Laboratory of Epigenetics and Metabolism, Institute for Molecular and Cellular Regulation, Gunma University, Gunma 371-8512, Japan; 9 Institute for Fundamental Biomedical Research, Johns Hopkins All Children’s Hospital, and Medicine in the Division of Endocrinology, Diabetes and Metabolism of the Johns Hopkins University School of Medicine, Petersburg, FL 33701, USA

**Keywords:** Cell biology, Cellular physiology, Pathophysiology

## Abstract

Mitochondria play a vital role in non-shivering thermogenesis in both brown and subcutaneous white adipose tissues (BAT and scWAT, respectively). However, specific regulatory mechanisms driving mitochondrial function in these tissues have been unclear. Here we demonstrate that prolonged activation of β-adrenergic signaling induces epigenetic modifications in scWAT, specifically targeting the enhancers for the mitochondria master regulator genes *Pgc1a/b*. This is mediated at least partially through JMJD1A, a histone demethylase that in response to β-adrenergic signals, facilitates H3K9 demethylation of the *Pgc1a/b* enhancers, promoting mitochondrial biogenesis and the formation of beige adipocytes. Disruption of demethylation activity of JMJD1A in mice impairs activation of *Pgc1a/b* driven mitochondrial biogenesis and limits scWAT beiging, contributing to reduced energy expenditure, obesity, insulin resistance, and metabolic disorders. Notably, JMJD1A demethylase activity is not required for *Pgc1a/b* dependent thermogenic capacity of BAT especially during acute cold stress, emphasizing the importance of scWAT thermogenesis in overall energy metabolism.

## Highlights

• JMJD1A catalytic activity is pivotal for mitochondrial biogenesis in beiging
• *Pgc-1* in white adipose is increased by JMJD1A-mediated histone demethylation
• Cold-induced *Pgc-1* levels in brown adipose is independent of histone demethylation
• JMJD1A catalytic activity protects from obesity and related metabolic disorders

## Graphical abstract

**Figure:**
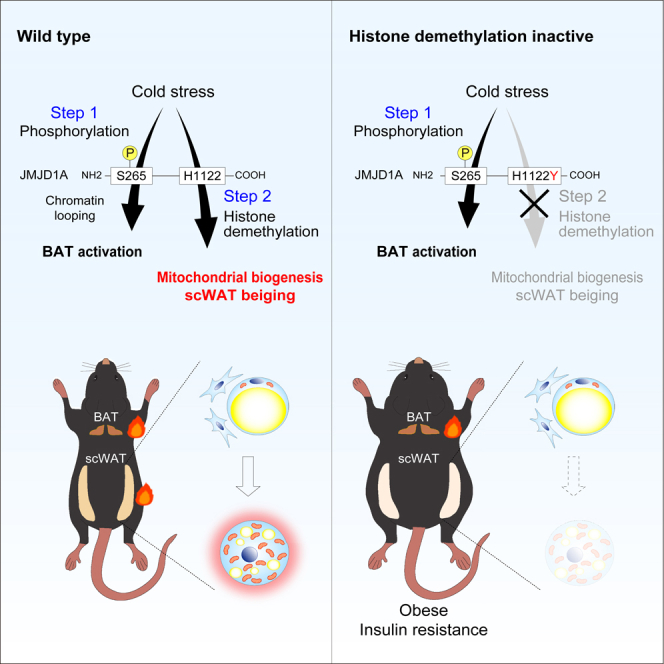

## Introduction

Obesity, characterized by excessive fat accumulation in adipose tissue, is a major risk factor for various comorbidities such as type 2 diabetes, dyslipidemia, hypertension, non-alcoholic hepatic steatosis, cardiovascular events, and certain type of cancers.^1^^,^^2^ Mammals have two functionally distinct adipose tissues: white adipose tissue (WAT) and brown adipose tissue (BAT).^3^^,^^4^ In particular, BAT serves as specialized tissue for dissipating excess energy through heat production. In response to cold stress-induced sympathetic activation, both BAT activation and conversion of subcutaneous white adipose tissue (scWAT) to a brown-like phenotype (called “beiging” of WAT) occur. Accordingly, uncoupling protein 1 (UCP1) expressed in the inner mitochondrial membrane, stimulates non-shivering thermogenesis in BAT and beige-WAT. However, beige adipocytes can also support thermogenesis independently of UCP1.^5^ This is of clinical importance as well because cold exposure in adult humans induces beige adipose tissue, presenting a promising route for dissipating excess calories as heat and offering a potential therapeutic strategy for age related metabolic diseases.^6^^,^^7^^,^^8^^,^^9^ Consequently, there is growing interest aimed at modifying mitochondrial function in adipose tissues to prevent metabolic diseases.^10^

Cells that consume high amounts of energy, such as brown adipocytes accumulate a high density of mitochondria during development. In contrast, white adipocytes, which are predominantly found in WAT and are responsible for energy storage, contain fewer mitochondria. However, under chronic cold stress, a population of white adipocytes undergo a transformation where they accumulate a high density of new mitochondria and transition into energy-consuming beige adipocytes.^11^ The process of mitochondrial biogenesis in BAT and beige adipocytes occur through similar but distinct processes that are tightly regulated by various transcriptional regulators, including peroxisome proliferator-activated receptor γ (PPARγ) coactivator 1α and 1β (PGC-1α and PGC-1β), nuclear respiratory factor 1 (NRF1) and GA binding protein transcription factor subunit α (GABPA, also known as nuclear respiratory factor 2, NRF2), and mitochondrial transcription factor A (TFAM). While the involvement of chromatin and its regulators in the dynamic changes in mitochondrial function in BAT and beige adipocytes is acknowledged, the similarities and differences between the two types of thermogenic adipocytes remains poorly understood; especially how epigenetic modifiers selectively affect mitochondrial biogenesis during the beiging process in response to activation by β-adrenergic receptor (β-AR) signaling.

Jumonji C-domain containing 1 A (JMJD1A), also known as JHDM2A or KDM3A, belongs to the Jumonji family of demethylases. It is responsible for removing mono- and di-methyl groups from lysine-9 of histone 3, a modification known as H3K9 methylation.^12^ JMJD1A plays a role in normal body weight control and adaptive thermogenesis, specifically through non-shivering thermogenesis in adipose tissue.^13^^,^^14^ It is activated through β-adrenergic receptor signaling that is activated upon environmental stress including cold exposure and excess calorie consumption. The activation of JMJD1A involves the phosphorylation of a specific serine residue at amino acid position 265.^15^ This phosphorylation event has distinct roles in the thermogenic roles in the two different types of thermogenic adipocytes that reside in either BAT or WAT.

The histone demethylation activity of JMJD1A is dispensable for its role in β-adrenergic dependent BAT activation. In this context, JMJD1A facilitates higher-order chromatin structural changes, promoting rapid gene transcription.^15^ However, in the process of WAT beiging, which involves a change in cellular identity, its histone demethylation activity is indispensable. This occurs in response to cold exposure where β-adrenergic stimulation phosphorylates JMJD1A at S265 and then it is recruited to thermogenic genes that are not yet fully accessible for transcription. JMJD1A then removes the repressive histone modification H3K9me2 from enhancer regions of thermogenic genes, enabling their expression.^14^ This two-step process involves signal sensing (step 1) followed by epigenomic reprogramming (step 2)^14^^,^^15^ (also reviewed in^16^^,^^17^^,^^18^).

Phosphorylation of JMJD1A at step 1 is also increased by inhibiting the MYPT1-PP1β phosphatase, which normally dephosphorylates JMJD1A,^19^ and this further promotes step 2 epigenomic reprogramming. This leads to enhanced beiging of scWAT which can potentially help alleviate obesity as well.^19^ On the other hand, mice with a specific mutation (serine 265 to alanine) that prevents phosphorylation of JMJD1A (*Jmjd1a*^SA/SA^ mice), results in both impaired BAT energy expenditure and WAT beiging due to defects in the signal sensing (step 1) and subsequent epigenetic rewriting (step 2). Overall, the phosphorylation and histone demethylation activities of JMJD1A play distinct roles in BAT activation and WAT beiging, highlighting its importance in regulating thermogenesis and cellular identity in response to cold and other stimuli.

Previous studies have shown that mice lacking *Jmjd1a* exhibit obesity, and metabolic disorders, but these effects could be attributed to a combination of impaired BAT activation and scWAT beiging^13^^,^^14^^,^^20^ because both *Jmjd1a*-null mice and *Jmjd1a*^SA/SA^ mice displayed defects in energy expenditure in both BAT and scWAT. This made it challenging to specifically investigate the role of H3K9 methylation in regulating cell identity in scWAT. To address this, we hypothesized that inactivating the catalytic activity of JMJD1A *in vivo* could specifically abolish the beiging process in scWAT without affecting the thermogenic capacity in BAT in response to cold stimuli. To test this hypothesis, mice with the H1122Y mutation (*Jmjd1a*^HY/HY^) were generated which results in a catalytic dead protein, allowing the investigation of the roles of temperature-dependent mitochondrial biogenesis and epigenomic reprogramming in regulation of beige adipogenesis.

## Results

### Cold-induced mitochondrial biogenesis in scWAT requires histone demethylation

**Figure 1:**
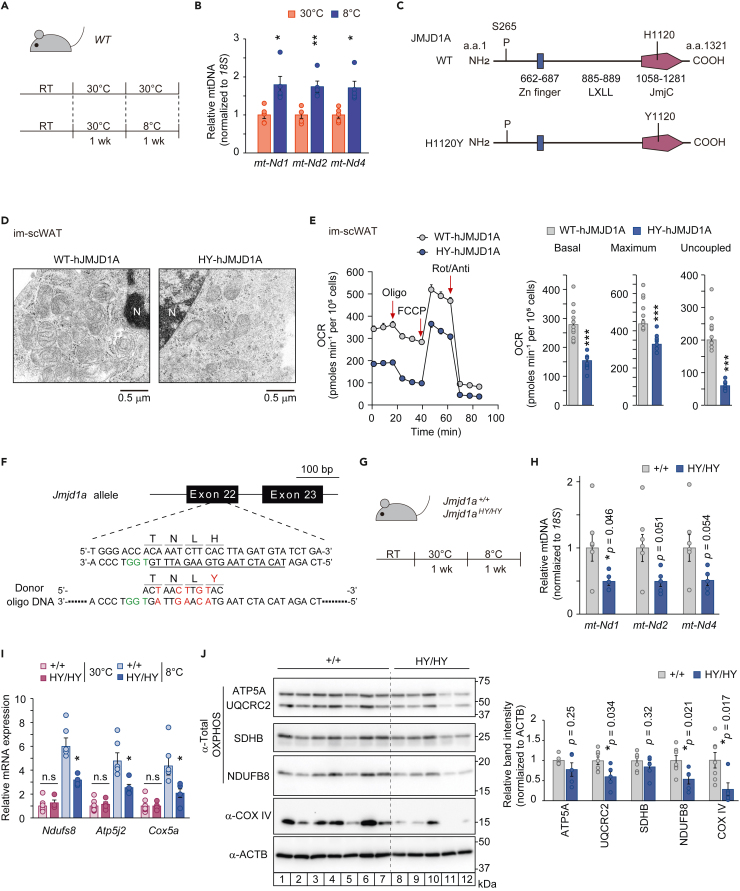
Cold induced mitochondrial biogenesis in scWAT requires histone demethylation (A) Schematic illustration of the chronic cold exposure experiment. C57BL6/J (wild-type*; WT*) mice were housed at 8°C or 30°C for 1 week after acclimation in thermoneutral conditions for 1 week. (B) Mitochondrial DNA content measured by qPCR using primers for *mt-Nd1*, *mt-Nd2* and *mt-Nd4* in the subcutaneous white adipose tissue (scWAT) of *WT* mice housed at 8°C (n = 5) or 30°C (n = 5) for 1 week. (C) Schematic representation of the domain structure of wild-type (*WT*) or H1120Y mutant hJMJD1A. (D) Representative transmission electron microscope images of differentiated im-scWAT cells expressing WT-hJMJD1A or HY-hJMJD1A (scale bar = 0.5 μm). N indicates nucleus. (E) Oxygen consumption rate (OCR) of immortalized (im)-scWAT cells overexpressing WT-hJMJD1A or HY-hJMJD1A, as measured by a metabolic flux assay. Arrows indicate the time of addition of oligomycin (Oligo), carbonyl cyanide 4-(trifluoromethoxy) phenylhydrazone (FCCP), and rotenone/antimycin A (Rot/Anti). Basal, maximum, and uncoupled respiration were calculated (mean ± SEM of five technical replicates). (F) Schematic diagram of the targeting strategy for the *Jmjd1a* H1122Y mutation using the CRISPR/Cas9 system. The gRNA-targeting sequence is underlined, the protospacer adjacent motif (PAM) sequence is colored green, and the mutated sequences of the donor oligo DNA are colored red. (G) Schematic illustration of the chronic cold exposure experiment. *Jmjd1a*^+/+^(+/+) and *Jmjd1a*^HY/HY^ (HY/HY) mice were housed at 8°C for 1 week after acclimation in thermoneutral conditions for 1 week. (H) Mitochondrial DNA content measured by qPCR using primers for the *mt*-*Nd1*, *mt-Nd2* and *mt-Nd4* genes in scWAT from *WT* (n = 7) and *Jmjd1a*^*HY/HY*^ (n = 5) mice exposed to chronic cold exposure (8°C) for 1 week. (I) The mRNA levels of *Ndufs8*, *Atp5j2* and *Cox5a* were determined by qPCR of scWAT from *Jmjd1a*^+/+^ or *Jmjd1a*^HY/HY^ mice with or without chronic cold exposure (8°C) for 1 week. (J) Immunoblot analysis of oxidative phosphorylation (OXPHOS) proteins, COX-IV, ATP5A, UQCRC2, SDHB, and NDUFB8, in tissue homogenates of scWAT from *Jmjd1a*^+/+^ (n = 7) and *Jmjd1a*^HY/HY^ mice (n = 5) with chronic cold exposure (8°C) for 1 week (left); band intensities were calculated using ImageJ software (right). Data are expressed as mean ± SEM (B, E, H, I, J). Representative of two independent experiments (E). Welch’s *t* test (B, E, H, I, J) was performed for comparison. ∗p < 0.05, ∗∗p < 0.01, and ∗∗∗p < 0.001 were considered statistically significant. n.s not significant. See also Figure S1. The uncropped images of the blots are shown in Figure S9.

To investigate the impact of cold exposure on mitochondrial biogenesis in scWAT, wild type mice were housed at either thermoneutrality (TN) (30°C) or cold temperature (8°C) for a week following an initial acclimation period at 30°C (Figure 1A). During the acclimation phase at 30°C, where β-adrenergic signaling is very low, residual beige adipocytes that are present at room temperature (RT, 23°C) disappear or undergo a process called whitening, leading to their transition into white adipocytes.^21^ This transition is associated with a decrease in thermogenic activity. In contrast, chronic exposure to cold, where β-adrenergic signaling is highly activated, promotes the robust generation of beige adipocytes from preadipocytes and/or white adipocytes through a process called beiging.^16^ These newly formed beige adipocytes undergo an increase in mitochondrial biogenesis that enhances thermogenesis. An increase in mitochondrial DNA (mtDNA) content was confirmed in the cold-exposure condition compared to the thermoneutral condition (Figure 1B) confirming an increase in mitochondrial mass. Additionally, the mRNA expression of thermogenic genes (*Ucp1* and *Elovl3*), nuclear-encoded mitochondrial genes (*Ndufs8* and *Cox5a*), the mtDNA-encoded gene (*mt-Nd3*) were all increased along with an increase in expression of key mitochondrial regulatory genes (*Pgc1a* and *Pgc1b*) in the cold exposed condition compared to thermoneutrality (Figure S1A).

Our previous studies documented two complementary roles for the H3K9me2 demethylase JMJD1A in adipose thermogenesis in BAT and scWAT. One requires the enzyme activity and the other does not^14^^,^^15^ (also reviewed in^16^^,^^17^^,^^18^). These studies were facilitated by making a knock-in mouse with a single amino acid change converting S265 to an alanine which prevented JMJD1A from being phosphorylated.^14^ In order to explore the role of the demethylase activity further, we needed to first engineer a mouse with an amino acid change that eliminated its catalytic activity.

The histidine 1120 of the Jumonji domain is highly conserved and essential for binding to Fe (II) (Figures 1C and S1B).^22^ This is reported to be required for its demethylase activity.^12^ To verify the functional significance of this specific residue, a mutation was introduced, replacing H1120 with phenylalanine (H1120F). Substituting histidine with phenylalanine or tyrosine is a common approach to assess the significance of a histidine residue in enzyme function due to their structural and chemical similarities to histidine. The wild type and its mutant versions were subsequently expressed and purified from insect Sf9 cells.^15^ Notably, only the wild-type recombinant protein exhibited significant H3K9 demethylase activity (Figures S1C and S1D), highlighting the crucial role of histidine 1120 in facilitating the demethylation process.

Next, we examined the role of H1120 in a cellular assay system using immortalized scWAT cells where the endogenous mouse *Jmjd1a* was knocked down using shRNA. These cells were then transduced with either wild type hJMJD1A or a mutant form carrying a mutation converting the conserved histidine to a tyrosine residue (H1120Y) which would also be predicted to eliminate demethylase activity.^19^ The cells transduced with wild type hJMJD1A displayed a high density of mitochondria after differentiation, indicating successful mitochondrial biogenesis (Figure 1D). However, cells transduced with the H1120Y mutant exhibited a significantly lower number of mitochondria and a reduced oxygen consumption rate (OCR) compared to those transduced with WT-hJMJD1A (Figures 1D and 1E). Importantly, the transduction of H1120Y-hJMJD1A not only impaired mitochondrial biogenesis (Figure 1D), but also blunted induction of the key thermogenic gene *Ucp1*.^19^ These results suggest that histone demethylation mediated by JMJD1A is crucial for activation of thermogenic gene expression and mitochondrial biogenesis during the differentiation of cultured preadipocytes into functional beige adipocytes.

To elucidate the physiological significance of JMJD1A-mediated histone demethylation in mitochondrial biogenesis in scWAT in live animals, we generated two lines of mice carrying a demethylation-defective form of JMJD1A with a histidine-1122 to tyrosine (H1122Y, or HY) mutation, which corresponds to the H1120Y mutation in human JMJD1A (Figures 1F, S1E, and S1F). Genotyping analysis of the offspring from *Jmjd1a*^HY/+^ parents revealed the number of mice with different genotypes. In line #1, there were 149 *Jmjd1a*^+/+^ mice, 241 *Jmjd1a*^HY/+^ mice, and 54 *Jmjd1a*^HY/HY^ mice. In line #2, there were 65 *Jmjd1a*^+/+^ mice, 116 *Jmjd1a*^HY/+^ mice, and 18 *Jmjd1a*^HY/HY^ mice (Figure S1G). These results indicated that fewer *Jmjd1a*^HY/HY^ pups were obtained than the expected Mendelian ratio. Immunoblot analysis using a specific anti-JMJD1A antibody (Figure S1H) showed that the expression of JMJD1A protein in scWAT was comparable between *Jmjd1a*^+/+^ and *Jmjd1a*^HY/HY^ (Figure S1I). However, it was observed that obtaining *Jmjd1a*^HY/HY^ males in line #1 was more challenging compared to line #2. This difficulty in obtaining *Jmjd1a*^HY/HY^ male in line #1 could be attributed to a phenomenon known as sex reversal, which had been previously reported in *Jmjd1a* knock-out mice.^23^ Therefore, female mice from line #1 and male mice from line #2 were used in the subsequent experiments to ensure an adequate representation of the *Jmjd1a*^HY/HY^ genotype.

To determine the roles of JMJD1A demethylation activity in cold-induced mitochondrial biogenesis during beiging, mice were exposed to cold temperature for 1 week (Figure 1G). It was observed that *Jmjd1a*^HY/HY^ mice had significantly lower levels of mitochondrial DNA-encoded *mt-Nd1* gene compared to *Jmjd1a*^+/+^ mice after cold exposure (Figure 1H). Additionally, there was a decreasing trend in the levels of *mt-Nd2* and *mt-Nd4* genes in *Jmjd1a*^HY/HY^ mice compared to *Jmjd1a*^+/+^ mice after cold exposure (Figure 1H). Under thermoneutral condition, the mRNA expression of nuclear-encoded mitochondrial *Ndufs8*, *Atp5j2*, and *Cox5a* genes were comparable between *Jmjd1a*^+/+^ and *Jmjd1a*^HY/HY^ mice (Figure 1I). Upon cold exposure, these genes were significantly induced in *Jmjd1a*^+/+^ mice, indicating an activation of mitochondrial biogenesis. In contrast, the induction of these genes was significantly lower in *Jmjd1a*^HY/HY^ compared to *Jmjd1a*^+/+^ mice (Figure 1I). In addition, the levels of mitochondrial proteins, including UQCRC2, NDUFB8, and COXIV, were lower in scWAT of *Jmjd1a*^HY/HY^ mice compared to *Jmjd1a*^+/+^ mice (Figure 1J). These results collectively indicate that histone demethylation activity of JMJD1A is required for cold-induced mitochondrial biogenesis in scWAT *in vivo*.

### Cold-induced mitochondrial biogenesis regulated by JMJD1A facilitates energy expenditure

**Figure 2:**
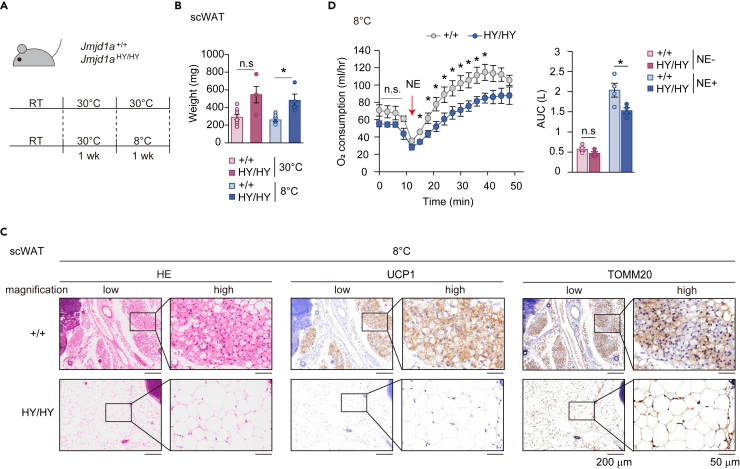
Cold-induced mitochondrial biogenesis mediated by JMJD1A increased energy expenditure (A) Schematic representation of chronic cold exposure experiment. *Jmjd1a*^+/+^ (+/+) and *Jmjd1a*^HY/HY^ (HY/HY) mice were housed at 8°C or 30°C for 1 week after acclimation in thermoneutral conditions for 1 week. (B) The weights of scWAT from *Jmjd1a*^+/+^ and *Jmjd1a*^HY/HY^ mice at 8°C or 30°C. (C) Hematoxylin and eosin (H&E), uncoupling protein 1 (UCP1) and TOMM20 staining of scWAT sections from *Jmjd1a*^+/+^ and *Jmjd1a*^HY/HY^ mice after cold exposure (8°C) (scale bar, 200 μm at low magnification or 50 μm at high magnification). (D) Norepinephrine (NE)-induced OCR in mice subjected to chronic cold exposure (8°C) for 2 weeks (*Jmjd1a*^+/+^: n = 4, *Jmjd1a*^HY/HY^: n = 4) (left). The OCR was analyzed before and 30 min after NE treatment (right). Data are the mean ± SEM (B, D). Welch’s *t* test (B) or repeated-measures ANOVA with a post-hoc Welch’s *t* test (D) were used for comparisons. ∗p < 0.05, considered statistically significant. n.s not significant. See also Figure S2.

To investigate the contribution of JMDJ1A-mediated mitochondrial biogenesis in scWAT to energy metabolism, *Jmjd1a*^+/+^ and *Jmjd1a*^HY/HY^ mice were acclimated to 30°C for 1 week and then housed at either 8°C or 30°C for an additional week as a control (Figure 2A). It was observed that *Jmjd1a*^HY/HY^ mice had higher scWAT weight compared to *Jmjd1a*^+/+^ mice at 8°C (Figure 2B). Body weights of *Jmjd1a*^+/+^ mice were increased by cold exposure, while those of *Jmjd1a*^HY/HY^ mice were unchanged. Gonadal WAT (gWAT) weights were similar between both genotypes at both 30°C and 8°C. On the other hand, BAT weights of *Jmjd1a*^+/+^ and *Jmjd1a*^HY/HY^ mice were increased by cold exposure suggesting that cold-induced BAT hyperplasia was not affected by the defect of JMJD1A catalytic activity (Figure S2A). Histological analysis of scWAT using hematoxylin & eosin (HE) staining and immunostaining for UCP1, a marker of thermogenic adipocytes, revealed that *Jmjd1a*^+/+^ mice developed abundant clusters of UCP1-positive adipocytes with multilocular lipid droplets specifically at 8°C, indicating the presence of thermogenic adipocytes (Figure 2C, left and middle panels). In contrast, *Jmjd1a*^HY/HY^ mice showed reduced level of UCP-1 positive adipocytes or multilocular lipid droplets in scWAT at 8°C suggesting impaired thermogenic capacity (Figures 2C, left and middle panels and S2B, left panel). Immunostaining for TOMM20, a mitochondrial marker, further supported these findings. *Jmjd1a*^*+/+*^ mice showed a strong signal in scWAT at 8°C, whereas scWAT from *Jmjd1a*^HY/HY^ mice showed a weaker TOMM20 signal (Figures 2C, right panels and S2B, right panel). This indicates there was a more robust increase in mitochondria in *Jmjd1a*^+/+^ vs. *Jmjd1a*^HY/HY^ mice. Importantly, the differences in scWAT phenotype between *Jmjd1a*^+/+^ and *Jmjd1a*^HY/HY^ mice were not observed under thermoneutral condition (Figures S2B and S2C). In gWAT, adipocyte sizes of *Jmjd1a*^HY/HY^ mice were significantly larger than those of *Jmjd1a*^+/+^ mice at 30°C, whereas at 8°C, there were no significant changes in the adipocyte size between the two genotypes (Figure S2D). These results indicate that JMJD1A-mediated mitochondrial biogenesis is specifically impaired under cold stress, resulting in a reduced presence of thermogenic adipocytes and compromised mitochondrial content in scWAT of *Jmjd1a*^HY/HY^ mice.

Cold-induced hyperplasia of BAT was not impaired by the absence of JMJD1A catalytic activity with the increased BAT weight upon chronic cold exposure in *Jmjd1a*^HY/HY^ mice (Figure S2A, right panel). Additionally, the UCP1 signals in BAT were comparable between *Jmjd1a*^+/+^ and *Jmjd1a*^HY/HY^ mice at 30°C, indicating similar basal thermogenic capacity (Figure S2E). Interestingly, upon exposure to 8°C for 1 week, both *Jmjd1a*^HY/HY^ and *Jmjd1a*^+/+^ mice exhibited similar levels of enhanced UCP1 signals in BAT, indicating the activation of thermogenesis in response to chronic cold stress was comparable. Under thermoneutral condition, BAT in *Jmjd1a*^HY/HY^ mice showed increased lipid accumulation compared to *Jmjd1a*^+/+^ mice. However, after chronic cold exposure, the levels of lipid droplets in BAT reached similar levels between the two genotypes (Figure S2E). This suggests that the differences in lipid accumulation in BAT between *Jmjd1a*^HY/HY^ and *Jmjd1a*^+/+^ mice at thermoneutrality are overcome upon chronic cold exposure, possibly due to the activation of thermogenic processes that promote lipid utilization. Furthermore, mRNA levels of genomic DNA-encoded mitochondrial genes, such as *Ndufs8* and *Sdhb*, as well as mitochondrial regulatory genes including *Pgc1a*, *Pgc1b*, in BAT showed no significant differences between two genotypes after chronic cold exposure (Figure S2F). This suggests that the transcriptional regulation of these mitochondrial genes and regulators in BAT during cold exposure is not affected by the JMJD1A histone demethylation activity.

**Figure 3:**
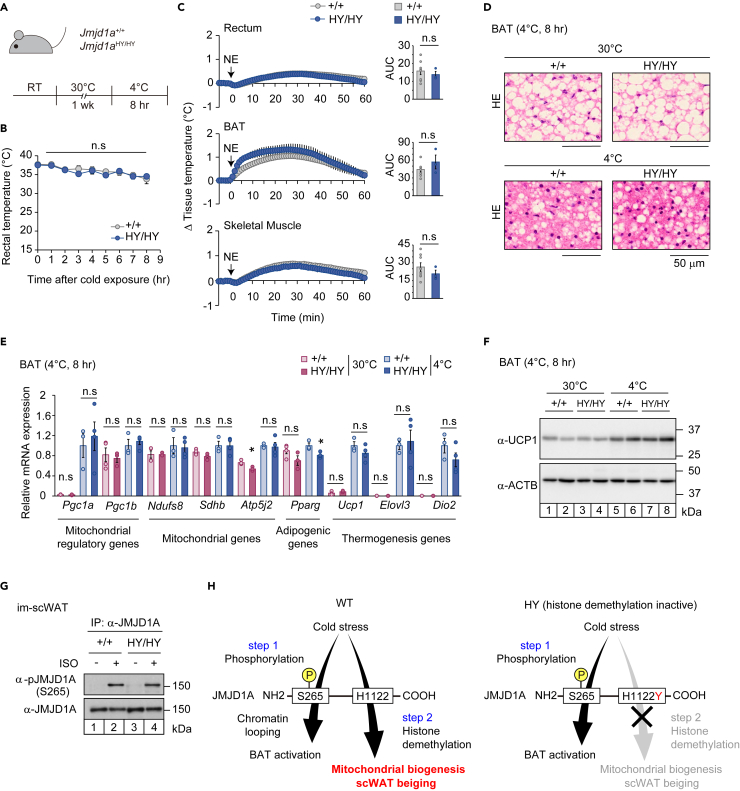
Thermogenic capacity in BAT is independent of JMJD1A demethylation activity (A) Schematic representation of the acute cold exposure experiment. *Jmjd1a*^+/+^ (+/+) and *Jmjd1a*^HY/HY^ (HY/HY) mice were exposed to cold at 4°C for 8 h after acclimation to thermoneutral conditions (30°C) for 1 week. (B) The rectal temperatures of 13-week-old *Jmjd1a*^+/+^ (n = 4) and *Jmjd1a*^HY/HY^ (n = 4) female mice were measured at the indicated times after cold exposure (4°C). (C) Real-time temperature changes (left) in the rectum (top), brown adipose tissue (BAT) (middle) and skeletal muscle on the back (bottom) of *Jmjd1a*^+/+^ (n = 8) and *Jmjd1a*^HY/HY^ (n = 3) mice. Arrows indicate the time point of intraperitoneal (i.p.) injection of 1 mg/kg BW NE. Cumulative AUC was calculated in each tissue (right). (D) Representative images of H&E staining of BAT from *Jmjd1a*^+/+^ and *Jmjd1a*^HY/HY^ mice at 30°C or 4°C (scale bar, 50 μm). (E) qPCR analysis revealed comparable expression of the mitochondrial regulatory genes *Pgc1a* and *Pgc1b*, the mitochondrial genes *Ndufs8*, *Sdhb* and *Atp5j2*, the adipogenic gene *Pparg*, and the thermogenic genes *Ucp1*, *Elovl3* and *Dio2* in the BAT of 22-week-old *Jmjd1a*^+/+^ (n = 7) and *Jmjd1a*^HY/HY^ (n = 7) mice under control or acute cold exposure (4°C for 8 h) conditions. (F) Immunoblot analysis of UCP1 in tissue homogenates of BAT from *Jmjd1a*^+/+^ and *Jmjd1a*^HY/HY^ mice. Actin was used as a loading control. (G) Immortalized scWAT cells from *Jmjd1a*^+/+^ and *Jmjd1a*^HY/HY^ mice were treated with 10 μM isoproterenol (ISO) for 20 min. Homogenates of these cells were subjected to immunoprecipitation (IP) with anti-mJMJD1A, followed by immunoblotting (IB) with anti-phospho-JMJD1A S265 or anti-mJMJD1A antibodies. (H) Model of adaptation to cold environment through JMJD1A phosphorylation and histone demethylation activity. In the acute phase, JMJD1A contributes to thermogenesis in BAT via phosphorylation and chromatin conformation changes. In the chronic phase, JMJD1A promotes thermogenesis through histone demethylation in scWAT. *Jmjd1a*^HY/HY^ mice have an impaired adaptive potential to chronic cold, although they retain the adaptability to acute cold. Data are the mean ± SEM (B, C, E). Repeated-measures ANOVA with a post-hoc Welch’s *t* test (B) or Welch’s *t* test (C, E) were performed for comparison. ∗p < 0.05 was considered statistically significant. n.s not significant. See also Figure S3. Uncropped images of the blots are shown in Figure S9.

To assess the impact of cold-induced mitochondrial biogenesis on whole body energy expenditure, we measured OCR in *Jmjd1a*^HY/HY^ and *Jmjd1a*^+/+^ mice. Norepinephrine (NE)-induced OCR after chronic cold exposure was significantly lower in *Jmjd1a*^HY/HY^ compared to *Jmjd1a*^+/+^ mice (Figure 2D). This suggests that the deficiency in JMJD1A histone demethylation activity impairs the ability of scWAT to increase energy expenditure in response to chronic cold exposure. In contrast, under thermoneutral condition, there was no significant difference in OCR between the two genotypes (Figure S2G). This indicates that the role of JMJD1A-mediated mitochondrial biogenesis in energy expenditure is specific to the chronic cold-induced activation of scWAT. Taken together, these results highlight the importance of JMJD1A histone demethylation activity in facilitating cold-induced mitochondrial biogenesis and enhancing energy expenditure specifically in scWAT. Because BAT induced thermogenesis was similar between the *Jmjd1a*^+/+^ and *Jmjd1a*^HY/HY^ mice, these results also demonstrate that scWAT beige conversion plays a significant role in whole body OCR (Figures 2B–2D, see below Figures 3A–3C).

### Demethylation activity of JMJD1A is dispensable for mitochondrial biogenesis and thermogenesis in BAT

The above findings indicate that the demethylation activity of JMJD1A is dispensable for mitochondrial biogenesis and thermogenesis in BAT. Because global *Jmjd1a*-deficient mice failed to maintain body temperature in acute phase of cold exposure,^14^^,^^15^ we assessed the thermogenic capacity and energy expenditure in BAT in *Jmjd1a*^+/+^ vs *Jmjd1a*^HY/HY^ mice. The mice were exposed to acute cold stress by keeping them at 4°C for 8 hours after one week of acclimation at 30°C (Figure 3A). Interestingly, we found that the body temperatures of *Jmjd1a*^HY/HY^ mice were similar to those of *Jmjd1a*^+/+^ mice throughout the 8 hour-cold exposure period (Figure 3B). We also measured the actual tissue temperature of BAT, as well as rectal and skeletal muscle temperature, and observed no significant differences between *Jmjd1a*^HY/HY^ and *Jmjd1a*^+/+^ mice after NE injection (Figure 3C). *Jmjd1a*^HY/HY^ mice have broader lipid droplet area in BAT than *Jmjd1a*^+/+^ mice at 30°C, while there was no difference in accumulated lipid between two genotypes at 8°C (Figures 3D and S3A). Furthermore, the expression of mitochondrial genes (*Ndufs8*, *Sdhb*, *Atp5j2*) and the adipogenic gene (*Pparg*) in BAT were comparable between the two genotype groups (Figure 3E). Importantly, mitochondrial regulatory genes (*Pgc1a*, *Pgc1b*) and thermogenesis genes (*Ucp1*, *Elovl3*, *Dio2*) were also similar in both genotypes under acute cold stress, indicating that demethylation activity of JMJD1A is not required for the induction of these genes in BAT (Figure 3E). Immunoblot analysis confirmed similar levels of induction of *Ucp1* expression after 8 h-cold exposure in both genotypes (Figures 3F and S3B). Also, the H1122Y mutation did not affect β-AR stimulated JMJD1A phosphorylation at S265, which is necessary for the catalytic-independent induction of thermogenic genes in BAT^14^^,^^15^ (Figure 3G). These results extend our previous studies using cultured brown adipocytes^14^^,^^15^ and demonstrate that the catalytic activity of JMJD1A is not necessary for the thermogenic function in mouse BAT in mice during acute cold exposure (Figure 3H).

### Chronic cold stress-induced *Pgc1* in scWAT requires histone demethylation of H3K9me2

**Figure 4:**
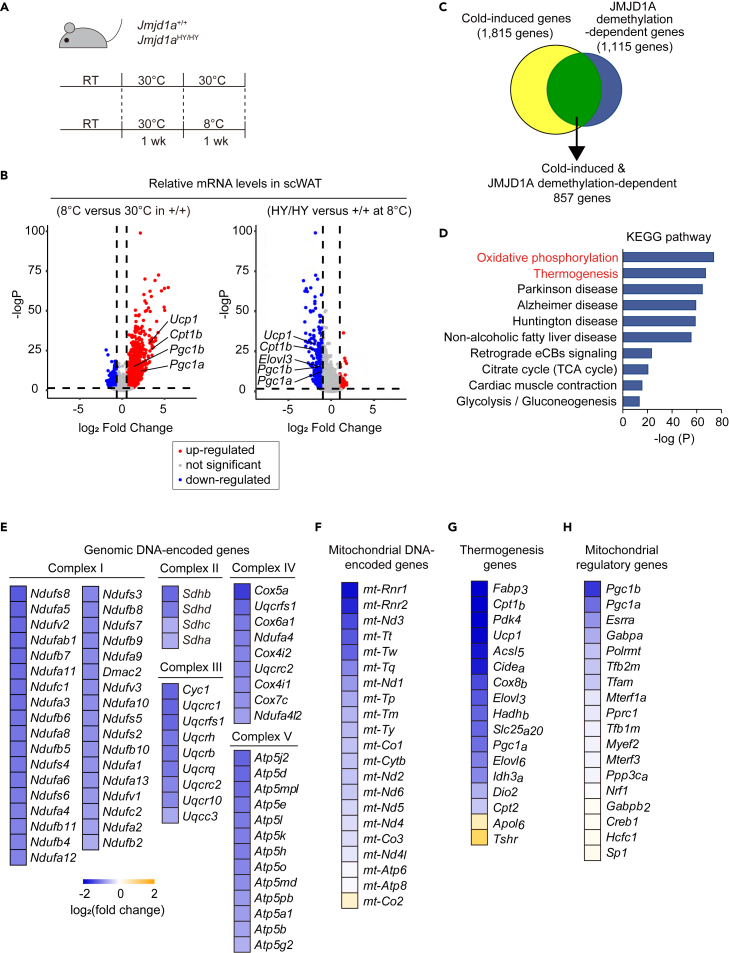
The demethylation activity of JMJD1A is required for the expression of thermogenesis and mitochondrial genes in response to chronic cold (A) Schematic illustration of the chronic cold exposure experiment. (B) Volcano plots comparing the gene expression in scWAT of *Jmjd1a*^+/+^ mice before and after cold exposure (8°C) (left) and scWAT of *Jmjd1a*^+/+^ and *Jmjd1a*^HY/HY^ mice after cold exposure (right). (C) Venn diagram showing the cold-induced genes (1,815 genes, defined in (B), left panel, up-regulated genes) and JMJD1A demethylation activity-dependent genes (1,115 genes, defined in (B), right panel, down-regulated genes). Overlapping genes were identified as JMJD1A demethylation-dependent cold-induced genes (857 genes). (D) Kyoto Encyclopedia of Genes and Genomes (KEGG) pathway analysis of 857 cold-induced JMJD1A demethylation-dependent genes defined in (C). (E) Heatmap showing the down-regulated genomic DNA-encoded genes, including the OXPHOS genes, determined by RNA-seq analysis of *Jmjd1a*^+/+^ or *Jmjd1a*^HY/HY^ scWAT at 30°C or 8°C. A color intensity scale is provided for reference. (F–H) Heatmap showing mitochondrial DNA-encoded genes (F), thermogenesis genes (G) and mitochondrial regulatory genes (H). Mitochondrial regulatory genes were defined as “mitochondrial gene expression”-related genes ways (https://www.wikipathways.org/). See also Figure S4.

To gain further insights into the impact of JMJD1A demethylation activity on thermogenesis, we conducted RNA-seq analysis using scWAT from *Jmjd1a*^HY/HY^ and wild-type mice exposed to either cold (8°C) or thermoneutral (30°C) conditions for one week (Figure 4A). Principal component analysis (PCA) clearly demonstrated the distinct transcriptome profiles for each group (Figure S4A). Analysis of differentially expressed genes in wild-type scWAT between 8°C and 30°C revealed 1,815 genes that were increased by cold exposure, including key beige-selective genes such as *Ucp1*, *Cidea*, and *Cpt1b*, which were defined as cold-induced genes (Figure 4B, left). Under cold condition, 1,115 genes were expressed at lower levels in *Jmjd1a*^HY/HY^ compared to wild-type mice, and these were defined as JMJD1A demethylation activity-dependent genes (Figure 4B, right). By comparing the genes from these two defined groups, we identified 857 cold-induced JMJD1A demethylation-dependent genes (Figure 4C). Pathway enrichment analysis showed that these genes were related to oxidative phosphorylation (OXPHOS) and thermogenesis (Figure 4D). Gene set enrichment analysis (GSEA) further supported these findings, indicating that inactivation of JMJD1A catalytic activity prevented the induction of thermogenesis and OXPHOS gene expression in scWAT under chronic cold exposure (Figures S4B and S4C). Specifically, the JMJD1A H1122Y mutation resulted in the loss of induction of OXPHOS genes, including both genomic DNA-encoded and mitochondrial DNA-encoded genes (Figures 4E, 4F, S4D, and S4E), as well as thermogenesis genes (Figure 4G).

In search for the regulatory genes that might be responsible for cold-induced mitochondrial biogenesis, we specifically examined regulatory genes associated with mitochondrial gene expression, as defined in WikiPathways. Our analysis revealed that inactivation of JMJD1A catalytic activity abolished induction of *Pgc1b*, *Pgc1a*, estrogen related receptor α (*Esrra*) and *Gabpa* expression in scWAT under cold conditions (Figure 4H). ESRRA and GABPA were known to regulate the transcription for mitochondrial genes through the DNA-binding and cooperation with PGC-1.^24^ These findings strongly suggest that demethylation activity of JMJD1A is crucial for the activation of *Pgc1* genes and the subsequent mitochondrial biogenesis during scWAT beiging in response to chronic cold exposure. Furthermore, we examined the expression of genes involved in mitochondrial fission and fusion, as these processes play a role in maintaining mitochondrial quality.^25^^,^^26^ However, we did not observe any differences in the mRNA expression of mitofission-related genes (*Drp1*, *Mff*, *Fis1*, and *Mief1*) or mitofusion-related genes (*Mfn1*, *Mfn2*, *Opa1* and *Oma1*) between *Jmjd1a*^+/+^ and *Jmjd1a*^HY/HY^ (Figures S4F and S4G). Taken together, these results indicate that the catalytic activity of JMJD1A specifically affects *Pgc1* function and mitochondrial biogenesis in scWAT beiging under chronic cold exposure, rather than influencing mitochondrial dynamics through fission and fusion processes.

### Cold-sensitive JMJD1A demethylates H3K9me2 in the enhancers of the mitochondrial master regulator *Pgc1,* inducing gene expression

To gain insight into the direct target genes of JMJD1A in beige adipocytes, we utilized previously published chromatin immunoprecipitation sequencing (ChIP-seq) data for JMJD1A in cultured beige adipocytes.^19^ This data was integrated with H3K27ac ChIP-seq data from beige adipocytes in scWAT under two conditions; exposure of mice to cold (referred to as cold beige) and following exposure to warm conditions after induction of beige adipocytes formation (referred to as warm beige).^21^ Initially, we classified H3K27ac peaks into 3 categories: cold beige-specific peaks (10,385 regions with fold change [FC] of H3K27ac cold beige/H3K27ac warm beige >2), warm beige-specific peaks (5,744 regions with an FC of cold beige H3K27ac/warm beige H3K27ac < 0.5), and common H3K27ac peaks (17,795 regions with an FC of cold beige H3K27ac/warm beige H3K27ac from 0.5 to 2) (Figure S5A). Next, we compared cold beige-specific H3K27ac peaks (10,385 peaks) with JMJD1A binding sites (39,364 peaks), and identified 2,089 overlapping peaks that were annotated to promoters of 1,647 genes (defined as 2 kb upstream to 1 kb downstream of TSS) (Figure S5B). Further comparison of the 857 genes cold-induced JMJD1A demethylation-dependent (as shown in Figure 4C) with 1,647 genes that exhibited both JMJD1A binding and cold beige-specific H3K27ac peaks lead to identification of 228 genes, including *Pgc1b* and *Pgc1a*. These 228 genes were defined as JMJD1A direct target genes, as they met the criteria of being cold-induced, JMJD1A demethylation-dependent, exhibiting cold beige-specific H3K27ac peaks, and having JMJD1A binding (Figure S5B; Table S1).

To identify putative enhancers of mitochondrial regulatory genes, particularly *Pgc1b*, that are epigenetically regulated under cold conditions, we conducted transposase-accessible chromatin sequencing (ATAC-seq) analysis using cultured beige adipocytes derived from *Jmjd1a*^+/+^ and *Jmjd1a*^HY/HY^. The ATAC-seq data revealed periodicity signals matching nucleosome units (Figure S6A) and their distribution along the transcription start site (TSS) (Figure S6B). Interestingly, the distribution of ATAC-seq peaks showed a similar pattern between *Jmjd1a*^+/+^ or *Jmjd1a*^HY/HY^ cells, with a higher proportion of peaks (27.2% for *Jmjd1a*^+/+^ and 22.3% for *Jmjd1a*^HY/HY^ cultured adipocytes, respectively) located at promoter-transcription start sites (TSS) among all the peaks (Figure S6C). This indicates that the chromatin accessibility profile, as indicated by the ATAC-seq peaks, was not significantly affected by loss of demethylation activity of JMJD1A (Figure S6C). By comparing open chromatin peaks in cultured preadipocytes and adipocytes that were differentiated into beige adipocytes in both genotypes, we identified total of 10,048 peaks in *Jmjd1a*^+/+^ and 12,500 in *Jmjd1a*^HY/HY^ cultured adipocytes. These peaks were specifically present in differentiated beige adipocytes (Figure S6D).

Gene ontology (GO) analysis of the 5,224 and 6,061 genes associated with the adipocyte differentiation-specific peaks for *Jmjd1a*^+/+^ (10,048 peaks) or *Jmjd1a*^HY/HY^ (12,500 peaks) respectively (Figure S6D) demonstrated an enrichment of cold-induced thermogenesis-related genes in adipocytes for both genotypes (Figure S6E). Moreover, we observed a significant enrichment of the EBF2, CEBP, and NFI transcription factor binding motifs within the identified peaks specific to beige adipocytes in both genotypes. This is significant because these factors are well-studied transcription factors involved in the induction of thermogenic genes^27^^,^^28^^,^^29^^,^^30^ (Figure S6F). These results indicate that our ATAC-seq data successfully captured regions of open chromatin that undergo dynamic changes during the differentiation of beige adipocytes.

**Figure 5:**
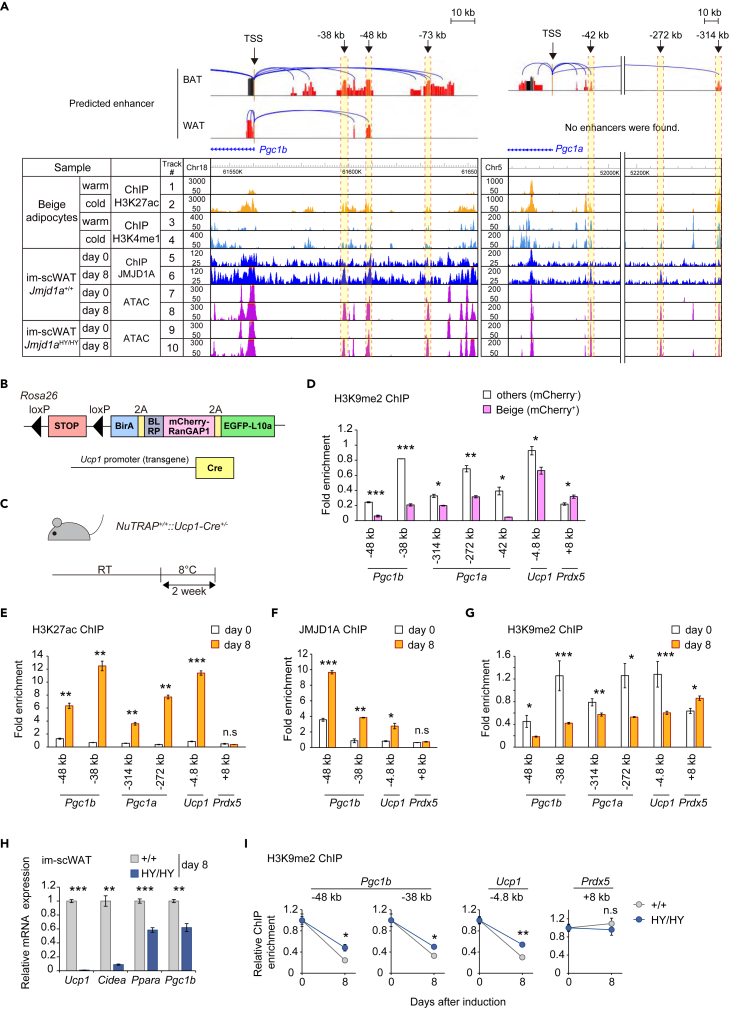
Histone demethylation in the enhancer region of mitochondrial regulator and thermogenesis genes by JMJD1A (A) Genome browser representation for H3K27ac, H3K4me1 in cold or warm beige adipocytes, and JMJD1A and ATAC-seq in im-scWAT cells on days 0 and 8 on *Pgc1b* or *Pgc1a* genomic regions. Distal enhancers of *Pgc1b* and *Pgc1a* predicted by Enhancer Atlas 2.0 are also presented on the genome browser. (B) Schematic representation of the transgene in NuTRAP::*Ucp1*-Cre mice. (C) NuTRAP::*Ucp1*-Cre mice were exposed to cold at 8°C for 2 weeks before analysis. (D) ChIP-qPCR analysis of H3K9me2 using sorted mCherry^+^ nuclei from beige adipocytes and mCherry^−^ nuclei from the other cells. (E–G) ChIP-qPCR analysis of H3K27ac (E), JMJD1A (F) and H3K9me2 (G) on indicated *Pgc1b*, *Pgc1a*, *Ucp1* and *Prdx5* enhancers during beige adipogenesis. (H) mRNA expression of indicated genes in differentiated im-scWAT cells (day 8) derived from *Jmjd1a*^+/+^ or *Jmjd1a*^HY/HY^ mice. (I) ChIP-qPCR showing the decrease in H3K9me2 levels on *Pgc1b* and *Ucp1* genes during beige adipogenesis, which was perturbed in the im-scWAT from *Jmjd1a*^HY/HY^ mice. Values of fold enrichment at day 0 of differentiation are set to 1. Data are the mean ± SEM (D–I). Representative of two (D–G) or three (H and I) independent experiments. Welch’s *t* test was performed for comparison in (D–I). ∗p < 0.05, ∗∗p < 0.01, and ∗∗∗p < 0.001 were considered statistically significant. See also Figures S5–S7.

To identify enhancer regions that are associated with beige specific genes influenced by both cold and thermoneutral temperatures, we integrated H3K27ac and H3K4me1 ChIP-seq dataset from beige adipocytes in scWAT of NuTRAP mice, a system allowing histone modification analysis from single cell types in animal tissues (explained in more detail below),^21^ with our ATAC-seq dataset from cultured beige adipocytes. This analysis revealed predicted enhancer regions of several genes, including *Pgc1b* (−38, −48, and −73 kb from transcription start site [TSS]), *Pgc1a* (−42, −272 and −314 kb) (mitochondrial regulatory genes) (Figure 5A), *Ucp1* (−4.8 kb), *Ppara* (−10 kb) and *Cidea* (−13.5 kb) (thermogenesis genes) (Figure S7A). Notably, the predicted enhancer regions for *Ucp1*, *Ppara* and *Cidea* were consistent with previous reports.^14^ To validate the predicted enhancer of *Pgc1b* and *Pgc1a*, we analyzed published HiC- data and confirmed that these enhancers (−38 and −48 kb of *Pgc1b* and −42, −272, and −314kb of *Pgc1a*) physically interacted with the TSS in 3T3-L1 adipocytes^31^ (Figure S7B). Furthermore, information in the database EnhancerAtlas also indicated that the distal regions of *Pgc1b* were annotated as enhancers in BAT^32^ (Figure 5A).

To assess the H3K9me2 levels of these differentiation regulated enhancers in beige adipocytes *in vivo*, we utilized NuTRAP::*Ucp1*-Cre mice, which allowed us to purify the nuclei specifically from beige adipocytes^21^^,^^33^ within a large background of white adipocytes. These mice expressed mCherry-labeled nuclei and EGFP-labeled ribosomes exclusively in beige adipocytes (Figure 5B). Following cold exposure, we isolated mCherry-labeled nuclei from WAT using flow cytometry (Figures 5C and S7C). Isolated nuclei were cross-linked, immunoprecipitated with anti-H3K9me2 antibody followed by ChIP-qPCR. The results indicated that the H3K9me2 levels at the enhancers of *Pgc1b* (−48 and −38 kb) and *Pgc1a* (−314, −272 and −42 kb) in mCherry-positive-beige cell nuclei were significantly reduced as compared to the H3K9me2 levels in mCherry-negative nuclei (non-beige cell nuclei) (Figure 5D), indicating that demethylation of H3K9me2 occurs in beige adipocytes in both *in vitro* and *in vivo* settings. These data also underscore that active epigenetic marks H3K27ac and H3K4me1 on *Pgc1a/b* enhancers overlapped with reduced H3K9me2 levels in beige adipocytes. Furthermore, the ChIP-qPCR analysis provided additional evidence of increased H3K27ac levels (Figures 5E and S7D) and recruitment of JMJD1A (Figure 5F) in the enhancers of *Pgc1b* and *Pgc1a*, while simultaneously showing a decrease in H3K9me2 levels to these regions during beige differentiation of cultured preadipocytes derived from scWAT (Figures 5G and S7E). Similar results were obtained for the *Ucp1*, *Ppara*, and *Cidea* enhancers. By contrast, there were minimal effects observed on histone modification or JMJD1A recruitment to the control region of *Prdx5* gene (Figures 5E–5G). Consistent with a key role for the enzyme activity of JMJD1A during adipocyte differentiation, we observed lower expression of *Ucp1*, *Cidea*, *Ppara*, and *Pgc1b* in differentiated im-scWAT adipocytes derived from *Jmjd1a*^HY/HY^ mice compared to *Jmjd1a*^+/+^ mice (Figure 5H). Additionally, H3K9me2 levels remained higher in *Jmjd1a*^HY/HY^ im-scWAT cells at the enhancers of *Pgc1b, Ucp1*, *Ppara*, and *Cidea* after beige adipocyte differentiation (Figures 5I and S7F). Interestingly, despite the lower expression and the higher histone methylation of these key adipogenic genes, ATAC-seq analysis from differentiated *Jmjd1a*^HY/HY^ im-scWAT cells before and after differentiation revealed an open chromatin state in these enhancers in both genotypes (Figure 5A, track 8 and 10), indicating that these enhancers are open upon differentiation and that H3K9me2 regulation occurs through changes in gene function not directly related to chromatin accessibility. The precise biological mechanism that H3K9me2 contributes to in this context remains unclear. However, these results indicate that the expression of *Pgc1* genes, key regulators of mitochondrial biogenesis, and other thermogenic genes are epigenetically controlled by JMJD1A during beige adipogenesis.

### Impaired mitochondrial biogenesis observed in *Jmjd1a*^HY/HY^ mice leads to obesity and metabolic disorders in aged mice

**Figure 6:**
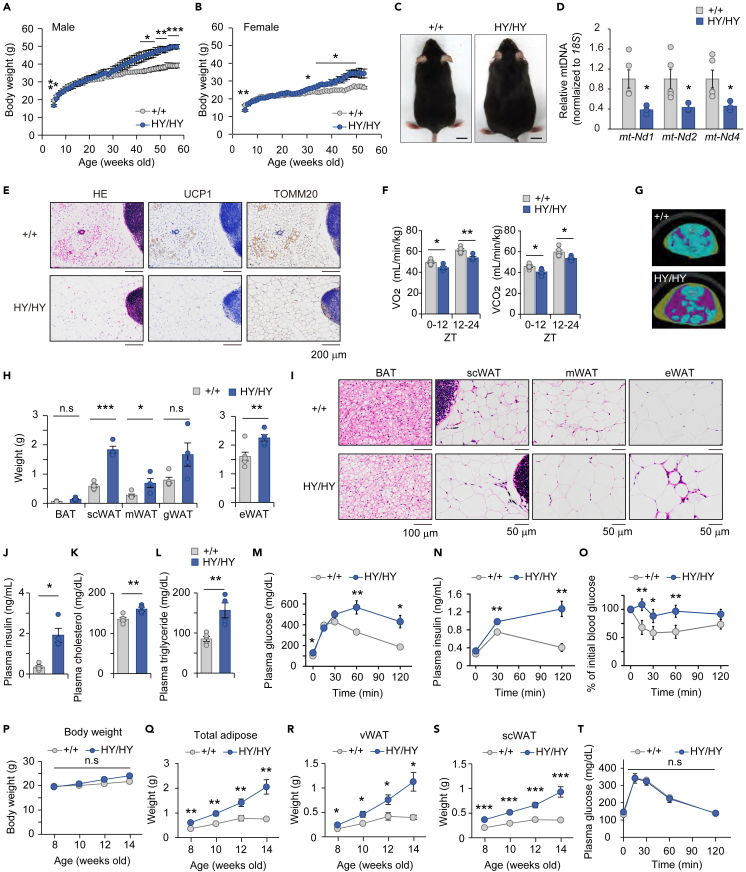
Impaired mitochondrial biogenesis causes obesity and metabolic dysfunction in *Jmjd1a*^HY/HY^ mice (A and B) Changes in body weight. *Jmjd1a*^+/+^ and *Jmjd1a*^HY/HY^ male mice (A, *Jmjd1a*^+/+^: n = 6, *Jmjd1a*^HY/HY^: n = 5) or female mice (B, *Jmjd1a*^+/+^: n = 5, *Jmjd1a*^HY/HY^: n = 4) were fed normal chow at 23°C. (C) Representative photographs of 53-week-old *Jmjd1a*^+/+^ and *Jmjd1a*^HY/HY^ female mice (scale bar, 10 mm). (D) Mitochondrial DNA content was measured by qPCR using primers for the *mt-Nd1*, *mt-Nd2,* and *mt-Nd4* genes in scWAT from 53-week-old *Jmjd1a*^+/+^ (n = 5) and *Jmjd1a*^HY/HY^ (n = 3) mice housed at 23°C. (E) Representative images of H&E and immunohistochemical staining for UCP1 and TOMM20 in scWAT sections from *Jmjd1a*^+/+^ and *Jmjd1a*^HY/HY^ mice (scale bar, 200 μm). Low magnification images are shown in Figure S8A. (F) The mean of oxygen consumption (VO_2_) and carbon dioxide production (VCO_2_) was calculated every 12 h. VO_2_ and VCO_2_ were normalized to mouse body weight (42 weeks old, *Jmjd1a*^+/+^: n = 5, *Jmjd1a*^HY/HY^: n = 4). (G) Representative computed tomography (CT) images of the lumber L4 vertebra of *Jmjd1a*^+/+^ and *Jmjd1a*^HY/HY^ female mice. Visceral fat, subcutaneous fat, and muscle are shown in purple, yellow, and blue, respectively. (H) Weights of BAT, scWAT, mesenteric (m)WAT, gonadal (g)WAT from female mice (53 weeks old, *Jmjd1a*^+/+^: n = 5, *Jmjd1a*^HY/HY^: n = 4), and epididymal (e)WAT from male mice (57 weeks old, *Jmjd1a*^+/+^: n = 6, *Jmjd1a*^HY/HY^: n = 5). (I) H&E stained sections of BAT (scale bar, 100 μm), scWAT, and mWAT from *Jmjd1a*^+/+^ and *Jmjd1a*^HY/HY^ female mice, and eWAT from male mice (scale bar, 50 μm). (J–L) Plasma insulin (J), cholesterol (K), and triglycerides (L) were measured in *Jmjd1a*^+/+^ and *Jmjd1a*^HY/HY^ mice in the fed state. (M and N) Glucose tolerance test (GTT). Plasma glucose (M) and insulin (N) concentrations were measured after the intraperitoneal injection of 2 g/kg glucose (49 weeks old, *Jmjd1a*^+/+^: n = 5, *Jmjd1a*^HY/HY^: n = 4). (O) Insulin tolerance test (ITT). Plasma glucose concentration was measured after the administration of 0.75 U/kg insulin (52 weeks old, *Jmjd1a*^+/+^: n = 5, *Jmjd1a*^HY/HY^: n = 4). (P–S) Changes in body weight (P) and fat mass determined by X-ray CT (Q-S) in 8- to 14- week-old *Jmjd1a*^+/+^ and *Jmjd1a*^HY/HY^ female mice (*Jmjd1a*^+/+^: n = 9, *Jmjd1a*^HY/HY^: n = 7). (T) GTT was conducted as described in (M) using 15-week-old female mice (*Jmjd1a*^+/+^: n = 11, *Jmjd1a*^HY/HY^: n = 4).Data are the mean ± SEM (A, B, D, F, H, J-T). Repeated-measures ANOVA with a post-hoc Welch’s – test (A, B, M-T) or Welch’s *t* test was performed for comparison in (D, F, H, J-L). ∗p < 0.05 and ∗∗p < 0.01, and ∗∗∗p < 0.001 were considered statistically significant. n.s indicates no significance. See also Figure S8.

Next, we followed male and female *Jmjd1a*^+/+^ and *Jmjd1a*^HY/HY^ mice over the course of a sixty-week time course at room temperature (23°C). It is important to note that this temperature is below the thermoneutral zone for mice, as evidenced by previous studies.^34^^,^^35^^,^^36^ Both genotypes of mice gained weight over time, however both male and female *Jmjd1a*^HY/HY^ gained more weight than the control starting at around 30 weeks of age (Figures 6A–6C). Because mice typically exhibit a partial cold-induced thermogenic response characterized by increased energy expenditure and activation of thermogenic adipocytes at 23°C relative to thermoneutrality, we hypothesized that the *Jmjd1a*^HY/HY^ mice may gain more weight because of a defect in mitochondrial biogenesis and beige associated thermogenesis. Consistent with this hypothesis, mitochondrial DNA qPCR analysis revealed that *Jmjd1a*^HY/HY^ mice had lower mitochondrial DNA content compared to *Jmjd1a*^+/+^ mice at 53 weeks of age (Figure 6D). Histological analysis showed that the *Jmjd1a*^*+/+*^ mice developed a small population of scWAT that express both UCP1 and TOMM20, whereas similarly aged *Jmjd1a*^HY/HY^ did not exhibit the same phenotype (Figures 6E and S8A). Indirect calorimetry analysis demonstrated that *Jmjd1a*^HY/HY^ mice had significantly lower oxygen consumption and carbon dioxide production during both light and dark phases as compared to *Jmjd1a*^*+/+*^ mice (Figures 6F and S8B, left and middle). These results suggest that mitochondrial biogenesis and beige adipocyte conversion in scWAT contributes to an increase in energy expenditure that significantly contributes to the management of body weight over time. However, there was no significant difference in respiratory quotient (RQ) between the two genotype groups, indicating that fuel substrates utilized by both genotypes were not significantly different (Figures S8B, right and S8C).

Analysis of body composition using X-ray computed tomography (CT) scanning revealed marked fat deposition in scWAT and visceral adipose tissue (vWAT) of *Jmjd1a*^HY/HY^ mice at 43 weeks of age (Figure 6G). Anatomical analysis confirmed that *Jmjd1a*^HY/HY^ mice had a greater body weight at the time of CT scanning (Figure S8D). Moreover, the total adipose tissue weight, including both vWAT and scWAT, in *Jmjd1a*^HY/HY^ mice was approximately 3.8 times higher compared to *Jmjd1a*^+/+^ mice (Figure S8E). Specifically, the weights of scWAT and vWAT, including mesenteric WAT (mWAT) and epididymal WAT (eWAT), were significantly higher in *Jmjd1a*^HY/HY^ mice compared to control mice (Figures 6H, S8F, and S8G). Female mice also showed an increasing trend in BAT and gWAT weights (Figures 6H, S8H, and S8I). Histological analysis further revealed lipid accumulation in BAT and adipocyte hypertrophy in scWAT, visceral mWAT, and gWAT in *Jmjd1a*^HY/HY^ mice (Figures 6I and S8J–S8L). Notably, the presence of crown-like structures (CLS) was observed in visceral eWAT of *Jmjd1a*^HY/HY^ mice, suggesting these mice also suffered from low grade chronic inflammation (Figure 6I). Consistent with this observation, the mRNA expression levels of inflammation-related genes, *Ccl2*, *Ccl5*, *Tnf-a*, *F4/80* and *Cd11b* in eWAT were significantly higher in *Jmjd1a*^HY/HY^ mice (Figure S8M). These findings suggest that impaired mitochondrial biogenesis in scWAT due to JMJD1A deficiency contributes to obesity, altered body composition, and possibly metabolic disorders.

In fact, the aged *Jmjd1a*^HY/HY^ mice had significantly higher levels of plasma insulin, cholesterol, and triglyceride compared to *Jmjd1a*^+/+^ mice (Figures 6J–6L). Additionally, *Jmjd1a*^HY/HY^ mice showed lower level of non-esterified fatty acid (NEFA) after fasting (Figure S8N). However, the levels of plasma glucose and total ketone bodies were comparable between the two genotype groups (Figures S8O and S8P). Plasma leptin, a hormone associated with satiety and adiposity, was approximately 3 times higher in *Jmjd1a*^HY/HY^ mice consistent with adipose tissue dysfunction (Figure S8Q). During the glucose tolerance test, *Jmjd1a*^HY/HY^ mice had higher plasma glucose and insulin levels compared to control mice (Figures 6M and 6N). Additionally, the insulin tolerance test showed higher plasma glucose levels in *Jmjd1a*^HY/HY^ mice compared to *Jmjd1a*^+/+^ mice (Figure 6O). These findings indicate that the glucose intolerance in *Jmjd1a*^HY/HY^ mice is attributable to insulin insensitivity. The presence of CLS in the visceral eWAT of *Jmjd1a*^HY/HY^ mice (Figure 6I) suggests that adipose tissue inflammation may contribute, at least in partially, to the insulin insensitivity observed in *Jmjd1a*^HY/HY^ mice. Based on these results, it can be concluded that *Jmjd1a*^HY/HY^ mice developed a range of biochemical abnormalities that are hallmarks of metabolic diseases that accumulate over time. The study highlights the importance of mitochondrial biogenesis in scWAT, especially in response to mild cold exposure (room temperature), as a protective mechanism against age related obesity and metabolic disorders.

To investigate if the metabolic abnormalities developed prior to the noticeable differences in body weight, we performed body composition measurements on young mice aged 8 to 14 weeks, which did not exhibit any body weight difference compared to *Jmjd1a*^+/+^ mice (Figure 6P). CT analysis revealed significantly higher adipose tissue content, including visceral and subcutaneous WAT, in *Jmjd1a*^HY/HY^ mice (Figures 6Q–6S). Notably, at 15 weeks of age, *Jmjd1a*^HY/HY^ mice showed similar glucose tolerance despite having larger adipose tissue compared to *Jmjd1a*^+/+^ mice (Figure 6T). These observations suggest that young *Jmjd1a*^HY/HY^ mice experience normal weight obesity and display metabolic abnormalities as they age. Overall, the findings of this study demonstrate that *Jmjd1a*^HY/HY^ mice exhibit a spectrum of metabolic abnormalities associated with aging, resembling characteristic features of metabolic diseases. These results underscore the crucial role of mitochondrial biogenesis in scWAT, particularly in response to mild cold exposure, as a protective mechanism against obesity and metabolic disorders. They also are consistent with the obesity/metabolic disease paradigm that excess body weight that occurs during aging precedes the appearance of significant metabolic disorders.

**Figure 7:**
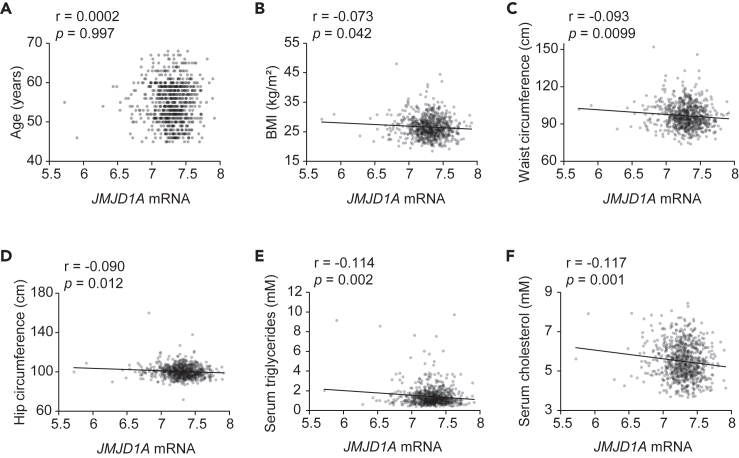
Correlation between the expression of *JMJD1A* and the risk of obesity and metabolic disorder in human cohorts (A–F) Correlation between the expression level of *JMJD1A* in human adipose tissue and age (A), BMI (B), waist circumference (C), hip circumference (D), serum triglycerides (E) and serum cholesterol (F). Pearson’s correlation coefficient was used to determine the correlation between *JMJD1A* expression level and the indicated parameters. These datasets were obtained from GSE70353.^37^^,^^38^

To further investigate the link between JMJD1A and metabolic disease, we utilized datasets of human metabolic parameters and adipose tissue gene expression from METSIM (METabolic Syndrome In Men), a cohort study of Finnish men.^37^^,^^38^ Our analysis revealed negatively correlation between *JMJD1A* expression and BMI, waist circumference, hip circumference, serum triglycerides, and serum cholesterol (Figures 7A–7F). This implies that JMJD1A may play a role in enhancing energy metabolism in adipose tissue and potentially preventing obesity in humans.

## Discussion

**Figure 8:**
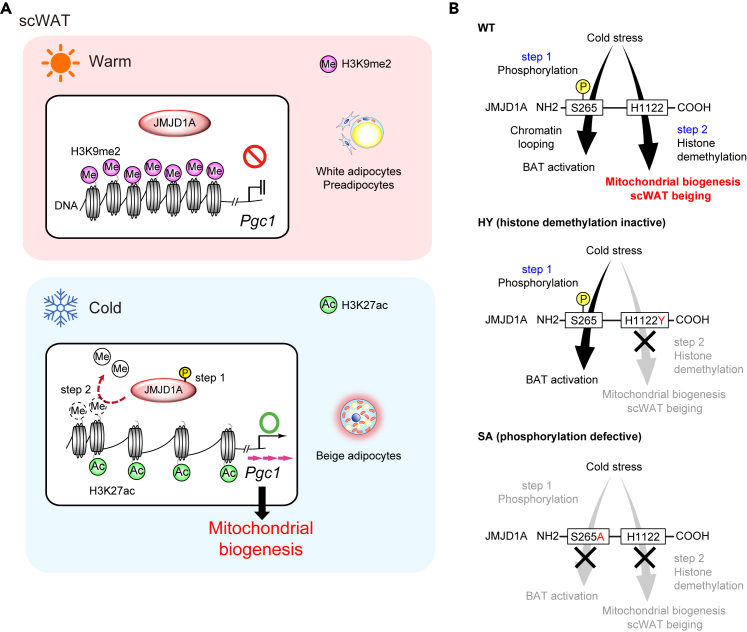
Summary of this study (A) Schematic model of cold-induced mitochondrial biogenesis via epigenetic reprogramming of mitochondrial regulatory genes during scWAT beiging by JMJD1A. (B) Distinct roles of serine phosphorylation (step 1) and histone demethylation (step 2) of JMJD1A in BAT activation and scWAT beiging. (Top) JMJD1A WT mice can activate BAT and induce WAT beiging via histone demethylation independent and dependent mechanisms. (Middle) JMJD1A demethylation inactive mice (HY) are unable to induce mitochondrial biogenesis in scWAT while BAT activation is unaffected. (Bottom) JMJD1A phosphorylation defective mice (SA) are unable to activate either BAT or scWAT beiging.

In our current study, we extend our previous studies^14^^,^^15^^,^^19^ to show that the expression of key mitochondrial regulators, such as *Pgc1a* and *b,* in scWAT are regulated by the histone demethylase JMJD1A in an external temperature-dependent manner in mice. We generated a mouse model with a single amino acid change in the Jumonji domain that eliminates iron binding which is critical to the α-ketoglutarate dependent demethylase activity of all Jumonji domain proteins. We used this *Jmjd1a*^HY/HY^ mouse model to demonstrate that JMJD1A plays a pivotal role in modulating H3K9me2 levels at enhancers of key mitochondrial genes such as *Pgc1a/b* to promote their expression, thereby facilitating mitochondrial biogenesis and the development of beige adipocytes during cold exposure in mice (Figure 8A). Our previous studies^14^^,^^15^ (also reviewed in^16^^,^^17^^,^^18^) combined with our current observations show that JMJD1A regulates thermogenesis in beige scWAT through a sequential two-step mechanism, starting with the phosphorylation of JMJD1A at amino acid S265, followed by the demethylation of histone H3K9, ultimately leading to the induction of mitochondrial biogenesis and beige adipogenesis. Notably, our data suggests that inactivation of JMJD1A’s catalytic activity does not influence chromatin opening (Figure S6C), suggesting that the demethylation of H3K9me2 promotes gene transcription by facilitating the recruitment of coactivators^19^ and other histone modifications, such as H3K9 acetylation to open sites.

Our study revealed that defects in JMJD1A-mediated demethylation is associated with obesity and metabolic disorders as mice age on a normal chow diet. Interestingly, young mice fed a normal diet showed no difference in body weight but had increased body fat mass (Figures 6Pand 6Q). These mice exhibited decreased scWAT beiging and thermogenesis due to reduced mitochondrial biosynthesis under mild cold exposure (room temperature). These phenotypes are recognized as “normal weight obesity” or “sarcopenic obesity” in humans which are linked to a higher risk of future metabolic abnormalities and insulin resistance.^39^ Mechanistically, our study emphasizes the critical role of JMJD1A in histone demethylation of key mitochondrial regulatory genes, PGC-1α and PGC-1β, for preventing obesity and metabolic abnormalities.

Aging is associated with a decline in mitochondrial quantity and function, contributing to the development of obesity over the life span in humans.^40^ Additionally, the activity of brown adipose and beige adipose tissues declines as humans age as well.^41^ This has sparked a growing interest in approaches to enhance mitochondrial function in adipose tissue to address and prevent obesity and metabolic diseases.^10^ The regulation of mitochondrial activity through histone demethylation holds promise as a potential avenue to achieve this objective.

JMJD1A also functions as a chromatin scaffold in brown adipocytes in BAT, which is independent of its enzyme activity.^15^ Importantly, our study further demonstrates that the absence of catalytic activity of JMJD1A does not influence brown adipocyte thermogenic functions, and thus, it has no impact on cold tolerance during acute cold stress (Figure 3), nor does it appear to be involved in the hyperplasia observed under chronic cold stress (Figures S2A and S2F). These findings indicate that JMJD1A’s contribution to acute thermogenesis in brown adipocytes is independent of histone demethylation activity. Instead, our previously reported PKA-induced JMJD1A phosphorylation and long-range chromatin looping, which bring active distal enhancers closer to the proximal promoter, play a more significant role in activating thermogenic genes such as *Ucp1*, *Dio2*, *Adrb1* and others, consistent with our previous reports.^13^^,^^14^^,^^15^^,^^19^ This critical result emphasizes the complementary roles played by JMJD1A in regulating thermogenesis in the two different types of thermogenic adipocytes, brown and beige.

Although JMJD1A catalytic activity has no impact on brown adipocyte thermogenic function, following the *Jmjd1a*^HY/HY^ over time also revealed a key role for JMJD1A in regulating body weight as mice age (Figure 6). The absence of JMJD1A’s catalytic activity led to impaired mitochondrial biogenesis in scWAT, resulting in increased weight gain that proceeded the development of metabolic abnormalities including glucose intolerance (Figure 6). These observations highlight the significance of JMJD1A in regulating epigenetic remodeling, promoting mitochondrial biogenesis in scWAT, which in turn prevents excess weight gain and glucose intolerance as mice age. These findings also provide significant support for the model that obesity associated metabolic disease is a consequence of excess weight gain.

Thermogenic adipocytes, brown adipocytes in BAT and beige adipocytes in scWAT, play a significant role in energy expenditure. Previous studies have highlighted the importance of thermogenesis in beige scWAT in maintaining systemic energy metabolism. For example, one study demonstrated that the loss of CD81, a marker of beige adipocyte progenitor cells, impairs the differentiation of beige adipocytes, but not brown adipocytes, leading to diet-induced obesity and insulin resistance.^42^ In contrast, the specific deficiency of PRDM16, a transcriptional regulator that promotes the differentiation of both brown and beige adipocytes, in brown adipocytes alone did not result in obesity.^43^ By elucidating the specific contribution of *Jmjd1a* in beige scWAT thermogenesis, our study provides additional evidence for the importance of beige adipocytes in maintaining energy balance and metabolic homeostasis.

Previous *in vivo* studies using *Jmjd1a* knock-in mice with a S265A mutation (preventing phosphorylation of JMJD1A) have shown reduced energy expenditure in both BAT and scWAT (Figure 8B),^14^ however, these mouse models were not sufficient for directly investigating the two-step regulatory mechanism mediated by JMJD1A *in vivo*. Therefore, we engineered a new complementary mouse model with a selective inactivation of the catalytic activity of JMJD1A, and results presented here provide more direct *in vivo* evidence supporting the involvement of the two-step mechanism in the induction of genes involved in mitochondrial biogenesis such as *Pgc1a* and *Pgc1b* in scWAT. Figure 8B presents an overview of the distinct yet overlapping phenotypes revealed by our studies using the three mouse models: step 1 signal sensing deficient mice (knock-in mice with a S265A mutation), step 2 epigenetic rewriting defective point mutant mice (knock-in mice with an H1122Y mutation). The combined studies expand our understanding beyond the regulation of beige-selective genes and highlights the broader role of JMJD1A in promoting mitochondrial biogenesis in response to β-AR signaling and in thermogenic regulation during cold exposure and aging.

In summary, our data highlight the importance of JMJD1A in epigenetic regulation of mitochondrial biogenesis in scWAT downstream of β-AR signaling. This process plays a critical role in regulating energy expenditure and also in preventing obesity associated metabolic disease as mice age. Thus, the concept of tissue-specific regulation of mitochondrial biogenesis through the rewritable epigenome provides a new perspective for the development of therapeutic interventions aimed at improving energy metabolism and combating metabolic disorders associated with obesity.

### Limitations of the study

Using *Jmjd1a*^HY/HY^ mice, we have shown that loss of JMJD1A activity in mice impairs both mitochondrial biogenesis and beige adipogenesis in scWAT, however, it is important to note that JMJD1A activity is globally inactivated in these mice. Therefore, it remains plausible that this effect is mediated by tissues and cell types beyond adipocytes, including potential influences from neuronal effects or immune cells. To address this possibility, tissue-specific deletion of JMJD1A is required in future studies.

## STAR★Methods

### Key resources table

**Table:** 

REAGENT or RESOURCE	SOURCE	IDENTIFIER
**Antibodies**		

mouse monoclonal anti-mouse JMJD1A antibody	Abe et al.^15^	IgG-F0618
mouse monoclonal anti-mouse JMJD1A antibody	Abe et al.^15^	IgG-F0231
mouse monoclonal anti-mouse JMJD1A antibody	This paper	IgG-F1313
rabbit polyclonal anti-phospho-JMJD1A (S265) antibody	Abe et al.^15^	#11890-2
mouse monoclonal anti-H3K27ac antibody	Kimura et al.^44^	CMA309/9E2H10
mouse monoclonal anti-H3K9me2 antibody	Hayashi-Takanaka et al.^45^	CMA317/6D11
rabbit polyclonal anti-UCP1 antibody	Abcam	ab10983; RRID:AB_2241462
total OXPHOS rodent WB antibody cocktail	Abcam	ab110413; RRID:AB_2629281
rabbit monoclonal anti-COX IV antibody	Cell Signaling Technology	4850T; RRID:AB_2085424
mouse monoclonal anti-ACTB antibody	Sigma-Aldrich	A5441; RRID:AB_476744
rabbit polyclonal anti-TOMM20 antibody	Proteintech	11802-1-AP; RRID:AB_2207530

**Bacterial and virus strains**		

pFastBac1-eXact-hJMJD1A-WT (489-1321)	Abe et al.^15^	N/A
pFastBac1-eXact-hJMJD1A-H1120F (489-1321)	This paper	N/A
pBabe SV40 large T antigen	Addgene	13970

**Chemicals, peptides, and recombinant proteins**		

Norepinephrine	Merck	A9512-250MG
Insulin	Sigma-Aldrich	I5523
Medetomidine	Nippon Zenyaku Kogyo	N/A
Midazolam	Maruishi Pharmaceutical	211-762100
Butorphanol	Meiji animal health	N/A
Antigen retrieval solution, pH 9.0	Nichirei Bio Sciences	415291
Collagenase D	Wako	034-22363
Dispase II	Thermo Fisher Scientific	17105-041
DMEM/Ham’s F12 medium	Sigma-Aldrich	D0547-10X1L
Fetal bovine serum	Thermo Fisher Scientific	10270
Penicillin and streptomycin sulfate	Nacalai Tesque	09367-34
Puromycin	Santacruz	sc-108071
Indomethacin	Sigma-Aldrich	I7378-5G
Dexamethasone	Sigma-Aldrich	D4902
3-isobutyl-1-methylxanthine	Sigma-Aldrich	I5879
Rosiglitazone	Wako	184-02651
T3	Sigma-Aldrich	T-2877
Oil red O	Wako	154-02072

**Critical commercial assays**		

Glucose C2 test kit	Wako	439-90901
NEFA C-test kit	Wako	279-75401
Autokit Total Ketone Bodies	Wako	415-73301
Triglyceride E-test kit	Wako	432-40201
Cholesterol E-test kit	Wako	439-17501
LBIS mouse insulin ELISA kit (U-type)	Wako	633-03411
Mouse leptin immunoassay kit	Morinaga Institute of Biological Science Inc.	M1305
Simple Stain Mouse MAX PRO	Nichirei Bio Sciences	414311
TruSeq RNA Sample Purification Kit	Illumina	RS-122-2001

**Deposited data**		

RNA-seq transcriptome data	This paper	GEO: GSE236733
ATAC-seq data	This paper	GEO: GSE236724
ChIP-seq data of JMJD1A	Abe et al.^14^	GEO: GSE107901
ChIP-seq data of H3K27ac and H3K4me1	Roh et al.^21^	GEO: GSE108077
HiC data	Siersbaek et al.^31^	GEO: GSE95533
Transcriptome of human adipose tissue	Civelek et al.^38^	GEO: GSE70353

**Experimental models: Cell lines**		

Sf9 cells	Thermo Fisher Scientific	11496015
im-scWAT cells transduced with WT-hJMJD1A	Takahashi et al.^19^	N/A
im-scWAT cells transduced with H1120Y-hJMJD1A	Takahashi et al.^19^	N/A
im-scWAT cells derived from *Jmjd1a*^+/+^ mice	This paper	N/A
im-scWAT cells derived from *Jmjd1a*^HY/HY^ mice	This paper	N/A

**Experimental models: Organisms/strains**		

C57BL/6J	Jackson laboratory Japan	N/A
*Jmjd1a*^HY/HY^ mice	This paper	N/A
NuTRAP mice	Jackson laboratory	029899
*Ucp1*-Cre mice	Jackson laboratory	024670

**Oligonucleotides**		

Primers used for Real-time PCR reactions	See Tables S3 and S4	N/A

**Software and algorithms**		

ImageJ	Schneider et al.^46^	https://imagej.net/ij/
IBM SPSS 28.0	IBM	N/A
FlowJo V10 software	BD Biosciences	N/A
FastQC	N/A	https://www.bioinformatics.babraham.ac.uk/projects/fastqc/
fastp	Chen et al.^47^	https://github.com/OpenGene/fastp
STAR	Dobin et al.^48^	https://github.com/alexdobin/STAR
StringTie	Pertea et al.^49^	https://ccb.jhu.edu/software/stringtie/
GFOLD	Feng et al.^50^	https://zhanglab.tongji.edu.cn/softwares/GFOLD/index.html
Trimmomatic	Bolger et al.^51^	http://www.usadellab.org/cms/?page=trimmomatic
Bowtie2	Langmead et al.^52^	https://sourceforge.net/projects/bowtie-bio/files/bowtie2/
SAMtools	Li et al.^53^	https://github.com/samtools/samtools
Picard	N/A	https://broadinstitute.github.io/picard/
deepTools	Ramirez et al.^54^	https://deeptools.readthedocs.io/en/develop/
HOMER	Heinz et al.^55^	http://homer.ucsd.edu/homer/
MACS3	Zhang et al.^56^	https://github.com/macs3-project/MACS
BEDTools	Quinlan et al.^65^	https://bedtools.readthedocs.io/en/latest/
Hicup	Lieberman-Aiden et al.^67^	https://www.bioinformatics.babraham.ac.uk/projects/hicup/
Juicer	Durand et al.^68^	https://github.com/aidenlab/juicer/

### Resource availability

#### Lead contact

Further information and requests for resources and reagents should be directed to and will be fulfilled by the Lead Contact, Juro Sakai (juro.sakai.b6@tohoku.ac.jp).

#### Materials availavility

Mouse lines generated in this study are available on a reasonable request from the lead contact, Juro Sakai (juro.sakai.b6@tohoku.ac.jp).

#### Data and code availability

• Data supporting this study are available upon request from the corresponding author. RNA-seq transcriptome and ATAC-seq data (days 0 and 8) have been deposited in the Gene Expression Omnibus (GEO) database with accession numbers GSE236733 and GSE236724, respectively. ChIP-seq data of JMJD1A were obtained from GSE107901, and ChIP-seq data of H3K27ac and H3K4me1 were obtained from GSE108077. HiC data were obtained from GSE95533. Datasets of human adipose tissue gene expression and metabolic parameters were obtained from GSE70353.
• This paper does not report original code.
• Any additional information required to reanalyze the data reported in this paper is available from the lead contact upon request.

### Experimental model and study participant details

#### Animals and Ethics Statement

All animal studies were conducted at Tohoku University and the University of Tokyo and adhered to the basic guidelines for conducting animal experiments established by the Animal Care and Use Committee. Mice were maintained on a chow diet (CE-2, CLEA Japan, Tokyo, Japan) with free access to water under a 12-h light/dark cycle at a constant temperature (22-24°C). The generation of *Jmjd1a*^HY/HY^ mice is described in detail below. The brown and beige adipocyte-specific studies involved crossing NuTRAP mice (Jackson Laboratory, 029899)^33^ with *Ucp1*-Cre mice (JAX024670).^57^ Both mouse lines, which were backcrossed for over eight generations with C57BL/6J mice, were generously gifted by Dr. Evan D. Rosen. Additional information regarding the age and sex of the mice is provided in Table S2.

#### Cell Culture

To isolate scWAT derived-stromal vascular fractions (SVFs), scWAT was dissected from 5-week-old *Jmjd1a*^+/+^ and *Jmjd1a*^HY/HY^ mice. The tissue was minced in phosphate buffered saline (PBS) and digested with collagenase D (Wako) and dispase II (Roche Diagnostics, Indianapolis, IN, USA) in PBS at 37°C for 40 min with shaking as described by Abe et al.^14^ Following digestion, the cell suspension was filtered through a 70-μm cell strainer into a new tube and then centrifuged at 800 × g for 5 min. The resulting cell pellet was resuspended in medium A, which consisted of Dulbecco’s Modified Eagle Medium (DMEM)/Ham's F-12 medium (Sigma-Aldrich) supplemented with 10% fetal bovine serum (Thermo Fisher Scientific, Waltham, MA, USA) and 100 units/ml penicillin and 100 μg/ml streptomycin sulfate (Nacalai Tesque, Kyoto, Japan). The cells were then cultured at 37°C with a 5% CO_2_ atmosphere in a humidified incubator. To immortalize cells, the cells were infected with a retrovirus expressing the large T antigen, pBabe SV40 large T antigen from Addgene (No. 13970). Three days after infection, the SVFs were selected using puromycin at a concentration of 0.5 μg/ml to establish immortalized cell lines referred to as im-scWAT cells. All the cells were confirmed to be free from mycoplasma infection using Mycoplasma Hoechst Stain Kit (MP biomedicals, Irvine, CA, USA).

For differentiation into beige adipocytes, im-scWAT cells were cultured to approximately 100% confluence. The cells were then treated with induction medium A containing 0.125mM indomethacin (Sigma-Aldrich), 5 μM dexamethasone (Sigma-Aldrich), 0.5mM 3-isobutyl-1-methylxanthine (Sigma-Aldrich), 0.5 μM rosiglitazone (Wako), 5 μg/ml insulin (Sigma-Aldrich), and 1 nM T3 (Sigma-Aldrich). After 2 days of induction, the cells were switched to medium A containing only insulin, T3, and rosiglitazone for another 6 days to support further differentiation and maturation into beige adipocytes.

For oil red O (ORO) staining, cells at specified stages of differentiation were rinsed with PBS and fixed in 3.7% formaldehyde in H_2_O for 10 min. After two washes with PBS and one wash with 60% isopropanol for 1 min, the cells were stained for 15 min in freshly diluted ORO solution (0.18% (wt/vol) ORO in 60% isopropanol). The stain was removed, and the cells were washed twice with PBS and photographed as previously described.^58^^,^^59^

### Method details

#### Antibodies

The mouse monoclonal antibody, immunoglobulin G F1313 (IgG-F1313) against mouse JMJD1A was produced by immunizing mice with affinity-purified recombinant full-length JMJD1A. Mouse monoclonal anti-mJMJD1A antibodies (IgG-F0618 and IgG-F0231), rabbit polyclonal anti-phospho-JMJD1A S265 (#11890-2),^15^ mouse monoclonal anti-H3K27ac IgG (CMA309/9E2H10),^44^ and mouse monoclonal anti-H3K9me2 IgG (CMA317/6D11)^45^ were developed as described previously. Antibodies against UCP1 (ab10983) and total OXPHOS rodent WB antibody cocktail (ab110413) were purchased from Abcam (Cambridge, UK). Anti-COX IV (4850T), anti-ACTB (A5441), and anti-TOMM20 (11802-1-AP) antibodies were obtained from Cell Signaling Technology (Danvers, MA, USA), Sigma-Aldrich (Saint Louis, MO, USA), and Proteintech (Rosemont, IL, USA), respectively.

#### Generation of *Jmjd1a*^HY/HY^ mice

To generate *Jmjd1a*^HY/HY^ mice, the following procedure was followed: a CRISPR-Cas9 expression vector (5 ng/μL), sgRNA targeting *Jmjd1a* exon 22 (5'- TACATCTAAGTGAAGATTTG -3'), and single-stranded donor DNA (10 ng/μL) containing the H1122Y mutation were injected into fertilized C57BL/6J eggs. The detailed method for harvesting fertilized eggs has been previously described.^60^ The injected single-cell embryos were transferred to pseudopregnant ICR mice. PCR-based screening and Sanger sequencing were performed to identify the founder mice. Genotyping was performed using clipped toes as the source of DNA. To amplify the *WT* allele, a specific set of primers (5'-GGAAATATGGGACCACAAATCTTC-3' for *WT* allele) and a reverse primer (5'-AGACAGTAAGCCCAGCTTCAA-3for both *WT* and *Jmjd1a*^HY^ allele) were used. To amplify the *Jmjd1a*^HY^ allele, a set of primers (5'-GGAAATATGGGACCACTAACTTGT-3' for *Jmjd1a*^HY^ allele), along with the common reverse primer for both the *WT* and *Jmjd1a*^HY^ allele, were used.

#### Cold exposure experiments

Mice were individually caged in a thermostatic chamber iB-230 (TAITEC, Aichi, Japan) and allowed to acclimate to thermal neutrality (30°C) for 1 week prior to the initiation of the cold exposure experiments. For acute cold exposure, the mice were transferred to different thermostatic chambers and maintained at 4°C for 8 h periods. For chronic cold exposure, the mice were placed in a chamber maintained at 8°C for 1 week.

#### Body temperature

Mice were restrained by hand, and a thermocouple RET-3 rectal probe (Physitemp, Clifton, NJ, USA) was gently inserted approximately 2 cm into the anus of the mice and held for 3 s to obtain the rectal temperature. The rectal temperature was monitored using a digital thermometer BDT-100 (BRC, Aichi, Japan) connected to the thermocouple RET-3 rectal probe.

#### Indirect calorimetry

Mice were placed in an individual acrylic metabolic chamber either set at RT or in a thermostatic chamber maintained at the indicated temperatures with unrestricted access to water and food and maintained on a 12-h light/dark cycle. Oxygen consumption (VO_2_) and carbon dioxide production (VCO_2_) were monitored using an O_2_/CO_2_ metabolic measurement system (MK-5000RQ; Muromachi Kikai, Japan). Measurements were taken every 3 min for 2 consecutive days. The respiratory quotient (RQ) was calculated as the ratio of VCO_2_ to VO_2_. The mean values of 240 measurements of the O_2_ and CO_2_ concentrations were used to calculate the total O_2_ consumption and CO_2_ production in the mice during each 12-h period.

To analyze the thermogenic function induced by norepinephrine (NE, Sigma-Aldrich), mice were intraperitoneally injected with NE (1 mg/kg body weight) and oxygen consumption was measured for 1 h using an indirect calorimeter (MK-5000RQ). Daily energy expenditure was also measured using an MK-5000RQ. The chamber volume was 720 ml, and the airflow to the chamber was 400 ml/min for mice acclimated to 30°C and 800 ml/min for mice acclimated to 8°C. Samples were collected every 3 min, and a standard gas reference was taken every 30 min.

#### Metabolite measurements

Plasma glucose levels were determined using the Glucose CII test (Wako, Osaka, Japan). Plasma non-esterified fatty acids (NEFA) and total ketone body levels were determined using the NEFA C test (Wako) and Autokit Total Ketone Bodies test (Wako), respectively. Plasma triglyceride and cholesterol levels were measured using the triglyceride E-test (Wako) and cholesterol E-test (Wako), respectively. Plasma insulin and leptin levels were determined using a mouse insulin ELISA kit (U-type, Wako) and a mouse leptin immunoassay kit (Morinaga Institute of Biological Science, Inc.), respectively, according to the manufacturer's instructions.

#### Glucose and insulin tolerance test

For the glucose tolerance tests (GTTs), mice were fasted overnight for 16 h, glucose (2 g/kg body weight) was administrated intraperitoneally, and the plasma glucose concentration was determined using the Glucose CII test (Wako). The plasma insulin concentration was measured during the GTT using an LBIS mouse insulin ELISA kit (U-type, Wako). For the insulin tolerance test (ITT), non-fasted mice were injected intraperitoneally with 0.75 U/kg insulin (Sigma-Aldrich) approximately 2 h after the start of the light cycle.

#### Computed tomography analysis

To measure the fat content of the mice, computed tomography (CT) was performed using a LaTheta LCT-200 (Hitachi-Aloka, Tokyo, Japan) system under anesthesia with 0.3 mg/kg medetomidine, 4 mg/mL midazolam, and 5 mg/mL butorphanol. To quantify the visceral and subcutaneous fat depots, the area between the proximal end of the lumbar vertebra L1 and the distal end of L6 was scanned. The weight of the fat depots was calculated using LaTheta software.

#### Histological analysis

Excised BAT and scWAT samples were fixed in 10% (v/v) neutral buffered formalin for 48 h at 4°C and embedded in paraffin. Immunostaining was performed on deparaffinized 3 μm sections, which were subsequently rehydrated, and the endogenous peroxidase activity was quenched via treatment with 0.3% hydrogen peroxide for 20 min. Antigen retrieval was performed by incubating the slides in an autoclave at 120°C for 5 min in antigen retrieval solution (pH 9.0; 415291, Nichirei Bio Sciences, Tokyo, Japan). Sections were incubated overnight at 4°C with rabbit anti-UCP1 antibody (ab10983, Abcam) or anti-TOMM20 antibody (11802-1-AP, Proteintech) at a dilution of 1:2000. UCP1 or TOMM20 signals were amplified with Simple Stain Mouse MAX PO (414311, Nichirei Bio Sciences), and the color was developed using a 3,3'Diaminobenzidine (DAB) substrate. The sections were counterstained with hematoxylin. Adipocytes were quantified with regards to area and number using the ImageRI_Adipocyte_Tools’ (https://dev.mri.cnrs.fr/projects/imagej-macros/wiki/Adipocytes_Tool) tool with a minimum size of 40 μm^2^ and a maximum size of 40000 μm^2^. More than 110 cells were counted in each individual during quantification.

#### Tissue temperature measurements

The mouse tissue temperature was measured according to a method described in a previous report.^61^ Briefly, anesthesia was induced in mice through the intraperitoneal injection of medetomidine hydrochloride (0.3 mg/kg), midazolam (4 mg/kg), and butorphanol (5 mg/kg). The mice were then placed on a self-regulating heating pad (NHP-M30N, Nissinrika, Tokyo, Japan) to maintain their rectal temperature at 37°C. To measure the tissue temperature, a thermocouple RET-3 rectal probe (Physitemp, Clifton, NJ, USA) was implanted into the rectum, whereas needle microprobes MT-29-1 (Physitemp, Clifton, NJ, USA) were inserted into the interscapular BAT and the skeletal muscle on the back. The tissue temperatures were recorded simultaneously using the TC-2000 Meter (Sable Systems International, North Las Vegas, NV, USA). Once the tissue temperature had stabilized for over 5 min, an intraperitoneal infusion of 1 mg/kg NE was administered. Changes in tissue temperature were continuously measured every second for 1 h, and the mean value per minute was calculated.

#### Transmission electron Microscopy

Differentiated cells expressing WT-hJMJD1A or HY-hJMJD1A were incubated in a solution containing 2% paraformaldehyde and 2.5% glutaraldehyde in 0.1 M cacodylate buffer (pH 7.4) for 1 h. The cells were then washed three times with 0.1 M cacodylate buffer. Subsequently, cells were treated with 1% OsO_4_ in 0.1 M cacodylate buffer for 15 min. After dehydration in a series of ethanol concentrations ranging from 50-100%, the dehydrated cells were embedded in Epon resin. Thin sections were cut using a microtome (EM UC-7; Leica, Wetzlar, Germany), stained with 2% uranyl acetate, 1% lead citrate, 1% lead acetate, and 1% lead nitrate, and images were obtained using a transmission electron microscope (JEM1400, JEOL, Tokyo, Japan).

#### Recombinant Protein purification

To purify the truncated form of the recombinant hJMJD1A-WT (amino acids [a.a.] 489–1321) and hJMJD1A-H1120F (a.a. 489-1321) proteins, a baculovirus expressing eXact™-tagged hJMJD1A was produced following a previously described method.^15^ To create pFastBac1-eXact-hJMJD1A-WT (489-1321) and pFastBac1-eXact-hJMJD1A-H1120F (489-1321) plasmids, fragments of the eXact™ tag sequence and either the WT or H1120F sequence of hJMJD1A (489-1321) were cloned into the pFastBac1 vector (Invitrogen). After infecting Sf9 cells with baculovirus-expressing eXact™-tagged WT or H1120F hJMJD1A (amino acids 489-1321), the harvested cell pellets were lysed using lysis buffer. Supernatants containing the lysed proteins were then incubated with Profinity eXact™ purification resin (Bio-Rad Laboratories, Hercules, CA, USA). After washing, the WT or H1120F hJMJD1A (489-1321) protein was eluted using elution buffer, as previous described.^15^

#### *In vitro* demethylation activity assay

To assess the demethylation activity of recombinant JMJD1A proteins, the HTRF-FRET demethylation assay was performed using a biotinylated dimethyl-histone H3K9me2 (1-21) peptide substrate (AnaSpec, Fremont, CA, USA) and a Europium cryptate-labeled anti-unmethylated histone H3K9 specific monoclonal antibody (anti-H3K9me0-Eu(K)) (Sceti Medical Labo, Tokyo, Japan), according to the manufacturer's protocol with slight modification as previously described.^15^

#### Immunoprecipitation and immunoblotting

Cultured cells were lysed in cell lysis buffer (50 mM HEPES-KOH [pH 7.9], 150 mM NaCl, 1.5 mM MgCl_2_ and 0.1% NP-40) and tissue homogenates were lysed in RIPA buffer (20 mM HEPES-KOH [pH 7.9], 1 mM EDTA, 150 mM NaCl, 1% Triton X-100, 0.1% SDS, and 0.5% DOC). Both buffers were supplemented with protease inhibitors (5 mg/ml pepstatin A, 10 mg/ml leupeptin, 2.8 mg/ml aprotinin, and 0.5 mM phenylmethylsulfonyl fluoride [PMSF]) and phosphatase inhibitors (40 mM NaF, 10 mM β-glycerophosphate, and 1 mM Na_3_VO_4_).

For immunoprecipitation with anti-JMJD1A antibody (IgG-F0618),^15^ 2 mg of the protein lysates were incubated with 3 μg of antibody. After a 2-h incubation at 4°C, Proteins G Sepharose 4 Fast Flow beads (GE Healthcare, Chicago, IL, USA) were added and incubated for another 1 h. The beads were washed three times with cell lysis buffer. Immunoprecipitates were subjected to immunoblotting using an anti-JMJD1A antibody (IgG-F0231)^15^ and anti-phospho-JMJD1A S265 (#11890-2).^15^ For immunoblotting, proteins were separated by sodium dodecyl sulfate-polyacrylamide gel electrophoresis, transferred to poly vinylidene di-fluoride (PVDF) membranes, and incubated with antibodies overnight at 4°C. Immunoblots were visualized by chemiluminescence using Pierce ECL Western Blotting Substrate (Thermo Fisher Scientific), and luminescence images were analyzed and quantified using ImageJ software.^46^

#### Evaluation of mtDNA copy number

The mitochondrial DNA (mtDNA) copy number was evaluated by determining the ratio of mtDNA to nuclear DNA (nDNA). mtDNA was extracted from scWAT using phenol/chloroform after digestion with proteinase K (150 μg/ml) in DNA lysis buffer (50 mM Tris-HCl [pH 7.9], 20 mM EDTA, 1% SDS, 100 mM NaCl) at 55°C overnight. The relative amounts of mtDNA were determined using quantitative real-time PCR relative to the 18S rRNA level. Mitochondrial coding genes encoding NADH dehydrogenase 1, 2, and 4 (*mt-Nd1, mt-Nd2,* and *mt-Nd4*, respectively) were selected to represent the mtDNA copy number.

#### Quantitative RT- PCR

Total RNA was extracted from cells using a Sepasol reagent (Nacalai Tesque) or from tissues using the ISOGEN reagent (Nippon Gene, Tokyo, Japan), according to the manufacturer's instructions. Extracted RNA (2 μg) was converted to cDNA using the SuperScript III First-Strand Synthesis System and oligo (dT) primers (Thermo Fisher Scientific). Real-time PCR was performed in 384-well plates using SYBR Green PCR buffer and the ABI PRISM 7900HT Sequence Detection System (Thermo Fisher Scientific). All reactions were performed in triplicate. mRNA expression is presented as a fold change relative to the indicated control after normalization to *Cyclophilin*^15^ or *Rpl32*.^62^ All primer sequences used in this study are listed in Table S3.

#### Chromatin immunoprecipitation assay

For ChIP using the anti-H3K27ac and anti-H3K9me2 antibodies, cells were cross-linked with 0.5% formaldehyde for 10 min at RT as previously described.^58^^,^^63^ For ChIP using the anti-JMJD1A antibodies, cells were cross-linked with 1.5mM ethylene glycol bis (succinimidyl succinate) (Thermo Fisher Scientific) for 30 min, followed by a second cross-linking with 0.5% formaldehyde for 10 min at RT, as previously described.^14^^,^^19^ Cross-linked cells were homogenized by passing through a 22 G needle 10 times in ice-cold hypotonic buffer (10 mM HEPES-KOH [pH 7.5], 1.5 mM MgCl_2_, 10 mM KCl, 1 mM EDTA, 1 mM EGTA) with protease inhibitors. Nuclear fractions were lysed in cell lysis buffer C (23 mM Tris-HCl [pH 8.0], 3.0 mM EDTA, 0.9% Triton X-100, 134mM NaCl, 0.2% SDS) with protease inhibitors, and fragmented to approximately 500 bp using a Branson SONIFIER 250 (Emerson, St. Louis, MO, USA). The sonicated nuclear fraction was incubated overnight with each antibody conjugated to Dynabeads Protein G (Thermo Fisher Scientific). After washing, decrosslinking, and elution, the immunoprecipitated DNA was purified using the QIAquick PCR purification kit (Qiagen, Venlo, Netherlands), and the concentration was determined using the Qubit double-stranded DNA High Sensitivity Assay Kit (Thermo Fisher Scientific).

The following antibodies were used for ChIP: mouse monoclonal anti-H3K27ac IgG (CMA309/9E2H10)^44^ (2 μg of antibody/30 μg of DNA), and mouse monoclonal anti-H3K9me2 IgG (CMA317/6D10)^45^ (2 μg of antibody/3 μg of DNA). All primer sequences used for ChIP-qPCR are listed in Table S4.

#### RNA-sequencing (RNA-seq)

RNA-seq libraries were prepared using the TruSeq RNA Sample Purification Kit (Illumina, San Diego, CA, USA), according to the manufacturer's protocol. Deep sequencing was performed on a HiSeq 2500 sequencer (Illumina, San Diego, CA, USA) using paired-end 50-base reads.

#### RNA-seq data analysis

For RNA-seq data analysis, FastQC (developed by the Bioinformatics Group at the Babraham Institute; https://www.bioinformatics.babraham.ac.uk/projects/fastqc/) was used to examine the quality of the sequencing reads from the FASTQ files. Adapter sequences and low quality bases were trimmed using fastp.^47^ After adapter trimming and filtering low-quality reads, the sequencing reads were aligned to the mm10 mouse genome using STAR.^48^ Fragments per kilobase of exon per million (FPKM) tables of RNA-seq data were calculated using StringTie^49^ with the GENOCODE comprehensive gene annotation set M25 Release. For the integrated analysis of RNA-seq and JMJD1A ChIP-seq data in cultured beige adipocytes^14^ (GSE107901), sequencing reads were aligned to the mouse genome mm9, and FPKM was calculated using GFOLD^50^ with the GENOCODE comprehensive gene annotation set M1 release.

#### ChIP-seq data analysis

For ChIP-seq data analysis, FastQC was used to examine the quality of sequencing reads in the FASTQ files. Adapter sequences and low-quality bases were trimmed using Trimmomatic.^51^ After adapter trimming and filtering of low-quality reads, sequencing reads were aligned to the mouse genome mm9 using Bowtie2^52^ for integrated analysis with databases, such as EnhancerAtlas.^32^ After the alignment of the ChIP-seq sequence reads, SAM files of the aligned sequences were converted to sorted BAM files using SAMtools.^53^ PCR duplicates were removed using Picard, developed by Broad Institute. Bigwig files were generated using the deepTools bamCoverage^54^ tool after removing signals from regions blacklisted by mm9.^64^ For peak calling, JMJD1A peaks were retrieved using the histone mode of the findPeaks function of HOMER,^55^ and the peaks of transcription factors were retrieved using MACS3.^56^ Overlapping peaks were defined as peaks with > 50% overlap and were calculated using the intersection function of BEDTools.^65^

#### ATAC-sequencing and data analysis

A nuclear suspension was prepared from im-scWAT cells derived from *Jmjd1a*^+/+^ or *Jmjd1a*^HY/HY^ mice, both before and after differentiation into beige adipocytes (day 0 or day 8), using a resuspension buffer (10 mM Tris-HCl, 10 mM NaCl, and 3 mM MgCl_2_, pH 7.4), as previously described.^66^ ATAC sequencing (ATAC-seq) was performed using the ATAC-Seq Kit (Active Motif, Carlsbad, CA, USA) according to the manufacturer's instructions (Active Motif). Briefly, 100,000 cell nuclei were incubated with the labeling mix containing Tn5 transposase at 37°C for 30 min. Subsequently, the DNA was purified and amplified using PCR. After purification, the library quality was assessed using a 4200 TapeStation system (Agilent Technologies, Santa Clara, CA). The library was sequenced using a NovaSeq 6000 system (Illumina, San Diego, CA, USA), generating approximately 30 million reads per sample. Data analyses included adaptor trimming, filtering, read mapping, sorting, PCR duplication removal, bigwig file generation, and peak calling. The same procedure was implemented for ChIP-seq data analysis. Fragment size visualization was performed using Picard, and metagene plots and heat maps were generated using BEDTools.^66^ Visualization of the genome-wide distribution and motif searching of ATAC peaks were performed using HOMER.^55^

#### Hi-C data analysis

Raw data from the Hi-C analysis of differentiating 3T3-L1 preadipocytes were obtained from a public database ( GSE95533).^31^ The data were processed using the Hicup^67^ and Juicer^68^ tools. Genomic interactions involving *Pgc1b* and *Pgc1a* were visualized using the Wash-U Epigenome Browser (https://epigenomegateway.wustl.edu/).

#### Nuclear isolation and sorting

Nuclear isolation was performed based on the method described by Roh et al..^21^^,^^33^ scWAT was homogenized using a digital homogenizer HK-1 (AS ONE, Osaka, Japan) in nucleus preparation buffer (NPB) containing 10 mM HEPES, 1.5 mM MgCl_2_, 10 mM KCl, 250 mM sucrose, 0.1% NP-40, and 0.2 mM DTT, and adjusted to pH 7.9. Homogenates were filtered through 100-μm cell strainers, cross-linked with 1% paraformaldehyde (PFA) at 25°C for 4 min, and then quenched by adding 125 mM glycine for 10 min. The cross-linked homogenates were centrifuged at 1000 × *g* for 10 min, and the nuclei-containing pellets were washed twice with NPB. Nuclear pellets were resuspended in nucleus sorting buffer (NSB) (10 mM Tris [pH 7.9], 40 mM NaCl, 90 mM KCl, 2 mM EDTA, 0.5 mM EGTA, 0.1% NP-40, 0.2 mM DTT), along with 20 ng/mL 4’,6-diamidino-2-phenylindole (DAPI) to label DNA. After filtering through 40-μm cell strainers, isolated nuclei were sorted using a FACS Aria II flow cytometer (BD, Franklin Lakes, NJ, USA), and gated by DAPI-stained DNA fluorescence. Singlets were gated using forward scatter (FSC), side scatter (SSC), and mCherry-positive and mCherry-negative populations. Data were analyzed using FlowJo v10 software (BD Biosciences, Franklin Lakes, NJ, USA). The collected samples were subjected to ChIP-qPCR using an anti-H3K9me2 antibody. Chromatin-immunoprecipitated DNA was subjected to qPCR using the SYBR green fluorescent dye.^19^^,^^58^^,^^63^

#### Extracellular flux measurement

Oxygen consumption rate (OCR) was measured using a Seahorse XF24 Extracellular Flux Analyzer (Seahorse Bioscience, North Billerica, MA, USA), as previously described.^14^^,^^15^^,^^19^ On day 7 of differentiation, cultured adipocytes were detached with trypsin (0.5 g/L)/EDTA (0.53mM) solution (Nacalai Tesque) and re-seeded at 1.0 × 10^5^ cells per well into XF24 V7 cell culture microplates (Seahorse Bioscience) in culture medium A (DMEM supplemented with 10% fetal bovine serum and penicillin/streptomycin). After 24 h, the cells were switched to a prewarmed assay medium (DMEM medium containing 25 mM glucose, 1 mM sodium pyruvate, and 2 mM L-glutamine). First, basal respiration was assessed in untreated cells. Second, ATP turnover was calculated in response to exposure to 4.4 μg/ml oligomycin (Oligo) (Wako). Third, maximum respiratory capacity was assessed after stimulation with 2.5 μM carbonyl cyanide 4-(trifluoromethoxy) phenylhydrazone (FCCP) (Sigma-Aldrich). Finally, mitochondrial respiration was blocked by adding both 1 μM rotenone (Sigma-Aldrich) and 1 μM antimycin A (Wako), and the residual OCR was considered as the nonmitochondrial respiration. Proton leak was calculated by subtracting the ATP turnover and nonmitochondrial respiration components of basal respiration, as described previously.^14^^,^^15^^,^^19^

#### Human data analysis

The association between the expression of JMJD1A and metabolic disorders were analyzed using the database of METSIM study, which includes the transcriptome data and metabolic parameters of 770 Finnish men (GSE70353).^37^^,^^38^

### Quantification and statistical analysis

All values are expressed as mean ± standard error of the mean unless otherwise specified. For normality, we used Shapiro-Wilk tests when the number of cohorts is greater than six. When the data indicated non-normality, the significances were evaluated by non-parametric Mann-Whitney U test. Significant differences between mean values were evaluated using a two-tailed Welch's t-test (when two groups were analyzed) or repeated-measures ANOVA with a post-hoc Welch’s t-test (when comparing repeated measurements). Pearson’s correlation coefficient was used to determine correlation between the level of *JMJD1A* expression in subcutaneous adipose tissue and the metabolic parameters. IBM SPSS 28.0 software (IBM, Armonk, NY, USA) was used for the statistical analysis. Significance was considered as *p* < 0.05.
