# Supplementary material for: Mitochondrial biogenesis in white adipose tissue mediated by JMJD1A-PGC-1 axis limits age-related metabolic disease
Source: iScience. 2024 Mar 1;27(4):109398. doi: 10.1016/j.isci.2024.109398 (PMC10966194; doi:10.1016/j.isci.2024.109398)
Supplement: Document S1. Figures S1–S9 and Tables S1–S4 [file mmc1.pdf]

iScience, Volume *27*

Supplemental information

Mitochondrial biogenesis in white adipose tissue mediated by JMJD1A-PGC-1 axis limits age-related metabolic disease

Ryo Ito, Shiyu Xie, Myagmar Tumenjargal, Yuto Sugahara, Chaoran Yang, Hiroki Takahashi, Makoto Arai, Shin-Ichi Inoue, Aoi Uchida, Kenji Nakano, Hyunmi Choi, Ge Yang, Yanan Zhao, Rei Yamaguchi, Hitomi Jin, Hina Sagae, Youichiro Wada, Toshiya Tanaka, Hiroshi Kimura, Tatsuhiko Kodama, Hiroyuki Aburatani, Kazuhisa Takeda, Takeshi Inagaki, Timothy F. Osborne, Takeshi Yoneshiro, Yoshihiro Matsumura, and Juro Sakai

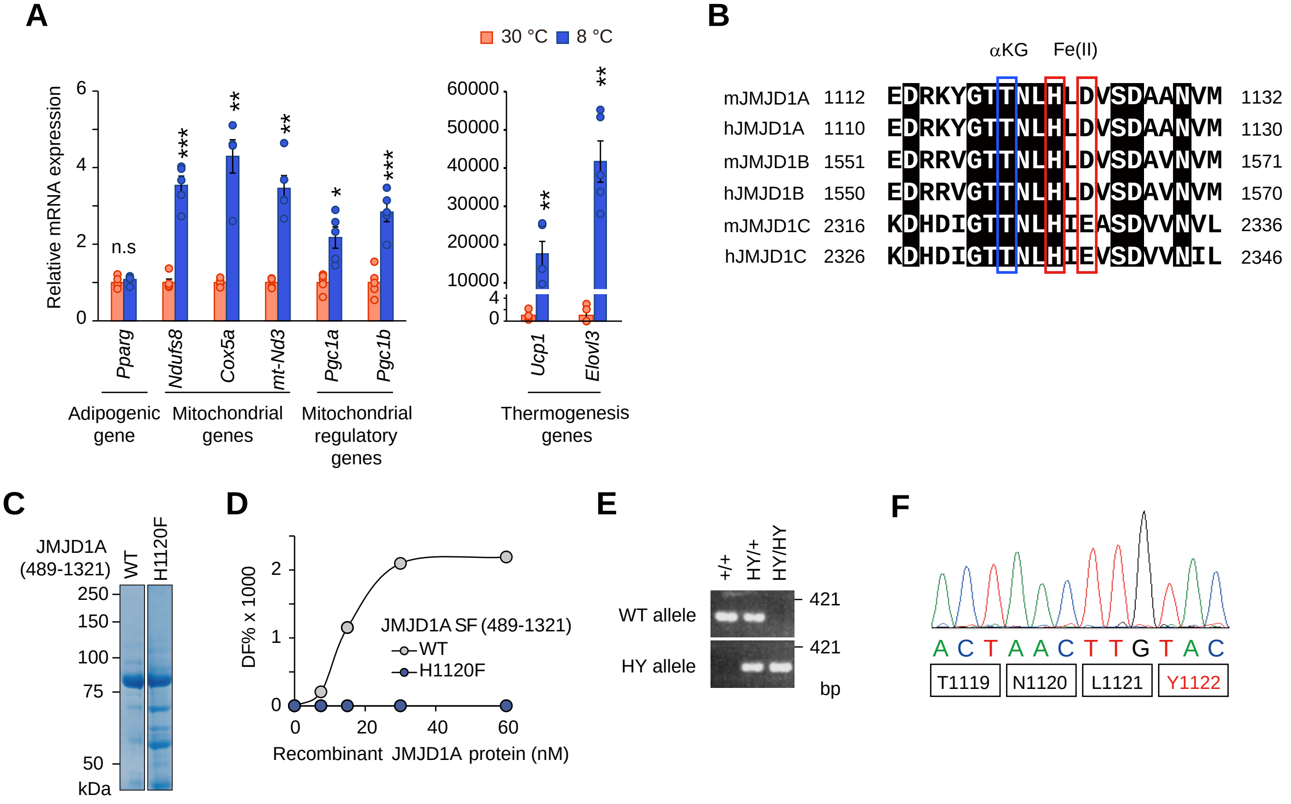

G

Line #1			
Genotype		Numbers (%)	
Jmjd1a	+/+	149	(34%)
Jmjd1a	HY/+	241	(54%)
Jmjd1a	HY/HY	54	(12%)
Total		444	(100%)

Line #2			
Genotype		Numbers (%)	
Jmjd1a	+/+	65	(33%)
Jmjd1a	HY/+	116	(58%)
Jmjd1a	HY/HY	18	(9%)
Total		199	(100%)

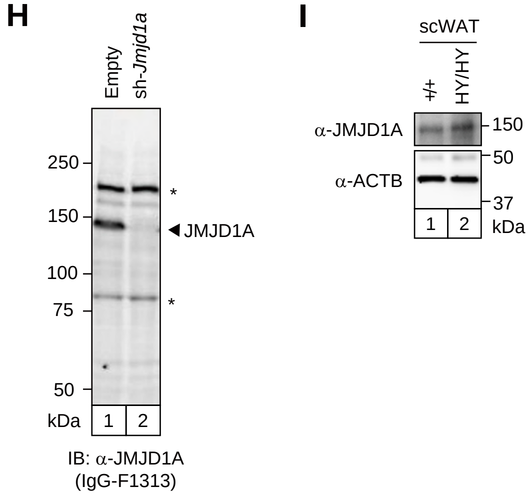

Figure S1. Generation of *Jmjd1a*$^{HY/HY}$ mice with loss of demethylation activity, related to Figure 1.
(A) mRNA expression measured by qPCR using primers for *Pparg*, *Ndufs8*, *Cox5a*, *mt-Nd3*, *Pgc1a*, *Pgc1b*, *Ucp1* and *Elovl3* in the scWAT of *WT* mice housed at 8ºC (n=5) or 30ºC (n=5) for a week.
(B) Alignment of amino acid sequences around α-KG and the Fe (II)-binding domain in the Jumonji C domain of mouse or human JMJD1 family members. Amino acids involved in α-KG and Fe (II) binding are indicated by blue and red squares, respectively.
(C) Recombinant human JMJD1A short-form (489-1321) proteins with or without the H1120F mutation were purified from Sf9 cells and subjected to SDS-PAGE and CBB staining.
(D) Histone demethylation activity of WT or H1120F JMJD1A (489-1321). Catalytic activities were examined using the HTRF-FRET histone demethylase assay, and the measured values are presented as DF% at the indicated concentrations of the recombinant JMJD1A protein (nM).
(E) Ethidium bromide-stained agarose gel showing PCR products for genotyping to distinguish *WT* (+/+), H1122Y/*WT* heterozygous (HY/+), and H1122Y homozygous (HY/HY) mice.
(F) Direct sequencing of genomic DNA from *Jmjd1a*$^{HY/HY}$ mice showing the tyrosine 1122 (TAC) mutation.
(G) Genotypes of the pups (n = 444 in line #1 and n = 199 in line #2) obtained by crossing *Jmjd1a*$^{HY/+}$ mice.
(H) Immunoblot analysis of JMJD1A in im-scWAT cells. Im-scWAT cells expressing control shRNA or shRNA targeting *Jmjd1a* were subjected to immunoblot analysis using an anti-mJMJD1A (IgG-F1313) antibody. Asterisks indicate non-specific bands.
(I) Immunoblot analysis of JMJD1A in scWAT from *Jmjd1a*$^{+/+}$ and *Jmjd1a*$^{HY/HY}$ mice.
Data are expressed as mean ± SEM (A). Welch's t test (A) was used for comparison. *$p < 0.05$, **$p < 0.01$, and ***$p < 0.001$ were considered statistically significant. The uncropped images of the blots are shown in Figure S9.

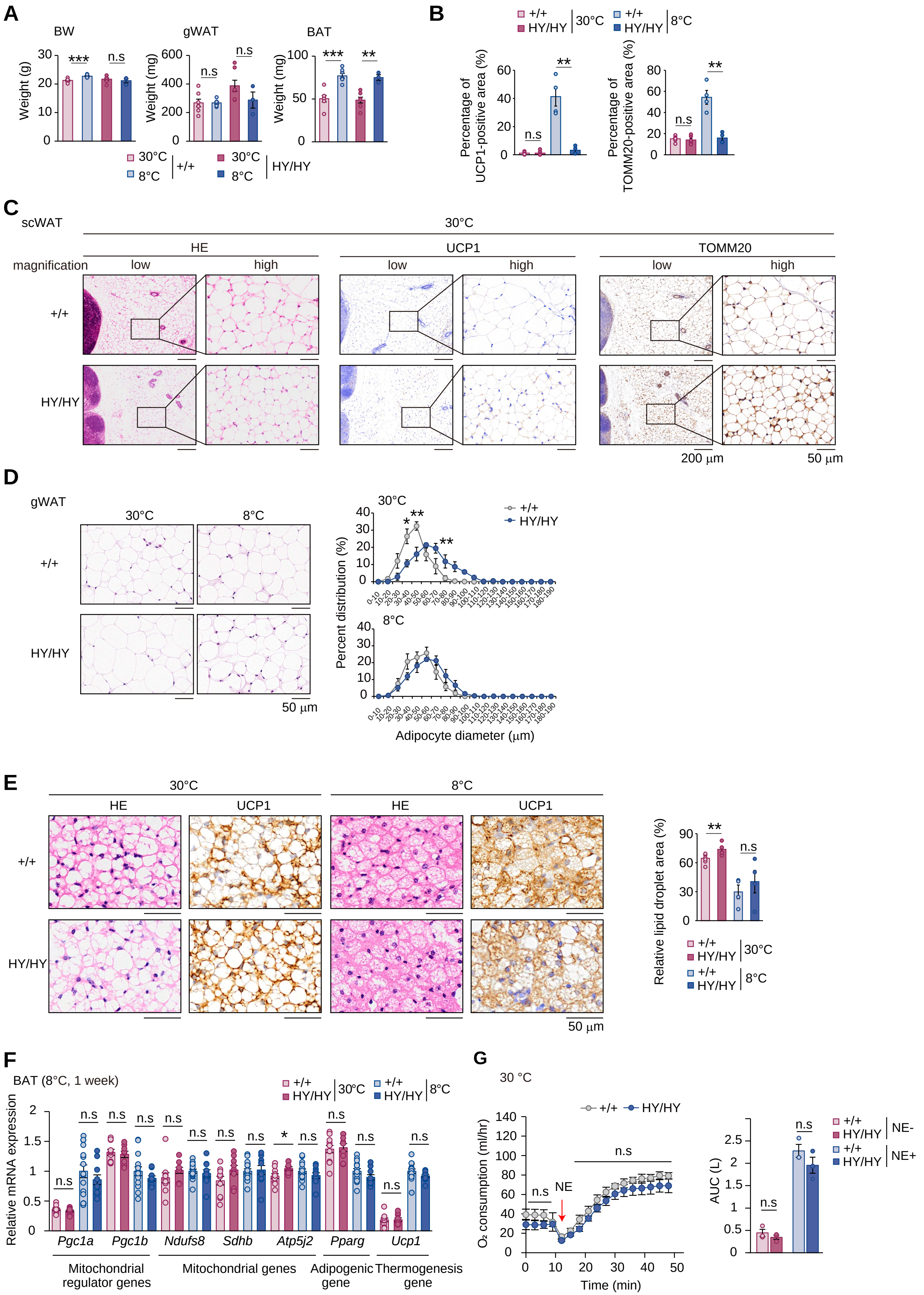

A
BW
gWAT
BAT
Weight (g)
Weight (mg)
***
n.s
**
30°C
8°C
+/+
HY/HY
B
Percentage of UCP1-positive area (%)
Percentage of TOMM20-positive area (%)
C
scWAT
30°C
HE
UCP1
TOMM20
magnification
low
high
200 μm
50 μm
D
gWAT
Percent distribution (%)
Adipocyte diameter (μm)
E
Relative lipid droplet area (%)
F
BAT (8°C, 1 week)
Relative mRNA expression
Pgc1a
Pgc1b
Ndufs8
Sdhb
Atp5j2
Pparg
Ucp1
Mitochondrial regulator genes
Mitochondrial genes
Adipogenic gene
Thermogenesis gene
G
30 °C
O2 consumption (ml/hr)
Time (min)
NE
AUC (L)
NE-
NE+

Figure S2. Cold-induced mitochondrial biogenesis mediated by JMJD1A promotes energy expenditure, related to Figure 2.

(A) The weights of total body, gWAT and BAT of *Jmjd1a*$^{+/+}$ and *Jmjd1a*$^{HY/HY}$ mice at 8ºC or 30ºC.

(B) Population of UCP1- or TOMM20-positive area was calculated per field of view in Figures 2C and S2C.

(C) H&E, UCP1, and TOMM20 staining of scWAT sections from *Jmjd1a*$^{+/+}$ and *Jmjd1a*$^{HY/HY}$ mice under thermoneutral conditions (scale bar, 200 μm at low magnification or 50 μm at high magnification).

(D) H&E staining of gWAT sections from *Jmjd1a*$^{+/+}$ and *Jmjd1a*$^{HY/HY}$ mice under thermoneutral conditions (30ºC) and chronic cold exposure (8ºC) (scale bar, 50 μm) (left). Line graph showing the distribution of adipocyte diameters in gWAT of *Jmjd1a*$^{+/+}$ and *Jmjd1a*$^{HY/HY}$ mice (right).

(E) Representative images of H&E and UCP1 staining of BAT sections from *Jmjd1a*$^{+/+}$ and *Jmjd1a*$^{HY/HY}$ mice under thermoneutral conditions (30ºC) and chronic cold exposure (8ºC) (left; scale bar, 50 μm). Relative lipid droplet area was calculated per field of view (right).

(F) mRNA levels of *Pgc1a*, *Pgc1b*, *Ndufs8*, *Sdhb*, *Atp5j2*, *Pparg* and *Ucp1* genes were determined by qPCR of BAT from *Jmjd1a*$^{+/+}$ or *Jmjd1a*$^{HY/HY}$ mice after chronic cold exposure (8ºC) or thermoneutrality (30ºC) conditions for 1 week.

(G) NE-induced OCR in mice housed in thermoneutrality (30ºC) for 2 weeks (*Jmjd1a*$^{+/+}$: n = 3, *Jmjd1a*$^{HY/HY}$: n = 3) (left). The OCR was analyzed before and 30 min after NE treatment (right).

Data are mean ± SEM (A, B, D-G). Welch’s t test (A, C-E, F (other than *Ndusf8* expression at 30ºC), G) and Mann-Whitney U test (F (*Ndusf8* expression at 30ºC)) were performed for comparison. *$p < 0.05$, **$p < 0.01$, and ***$p < 0.001$ were considered statistically significant. n.s not significant.

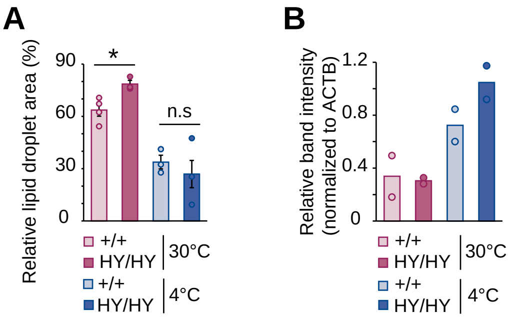

Figure S3. JMJD1A demethylation activity is dispensable for thermogenic capacity in BAT, related to Figure 3.

(A) Relative lipid droplet areas in the BAT of *Jmjd1a*$^{+/+}$ and *Jmjd1a*$^{HY/HY}$ mice were calculated per the field of view from the images of H&E staining as shown in Figure 3D.

(B) Signal intensities of the UCP1 immunoblots in Figure 3F were calculated using ImageJ software. Actin was used as a loading control.

Data are mean ± SEM (A) or mean (B). Welch's t test (A) was performed for comparison. $*p < 0.05$ was considered statistically significant. n.s not significant.

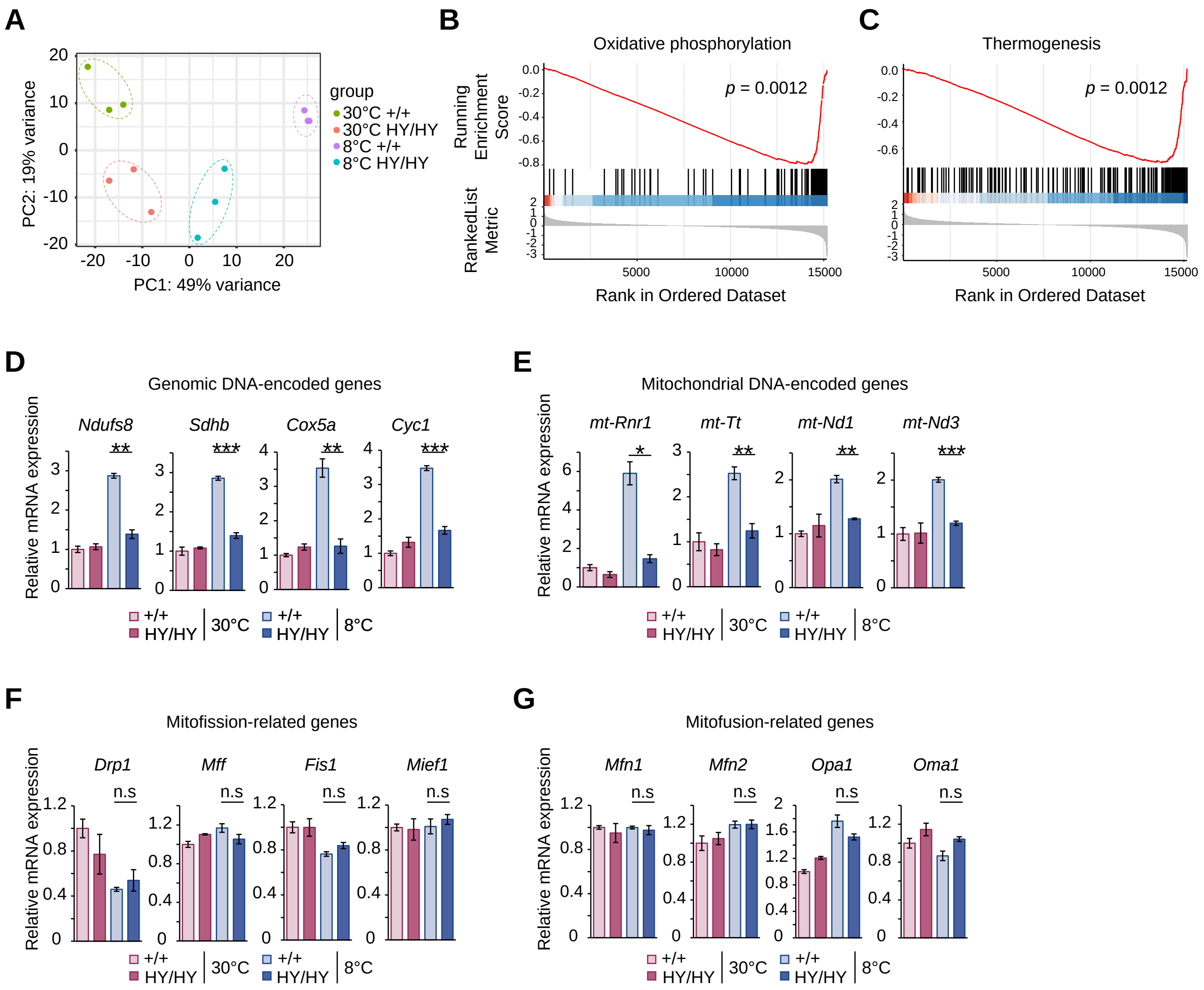

Figure S4. JMJD1A demethylation activity is needed for the expression of thermogenesis and mitochondrial genes in response to chronic cold, related to Figure 4.

(A) Principal component analysis (PCA) of all transcribed genes in scWAT of *Jmjd1a*$^{+/+}$ and *Jmjd1a*$^{HY/HY}$ mice at 8ºC or 30ºC.

(B and C) Gene set enrichment analysis (GSEA) identifying negatively enriched “oxidative phosphorylation” (B) and “thermogenesis” (C) of JMJD1A demethylation-dependent cold-induced genes.

(D-G) Relative gene expression calculated using FPKM values from RNA-seq data of genomic DNA-coded mitochondrial genes (D), mitochondrial DNA-encoded genes (E), mitofission-related genes (F), and mitofusion-related genes (G). These values were normalized to those of *Jmjd1a*$^{+/+}$ cells at 30ºC.

Data are mean ± SEM (D-G). Welch’s t test (D-G) was used for comparison. $*p < 0.05$, $**p < 0.01$ and $***p < 0.001$ were considered statistically significant. n.s not significant.

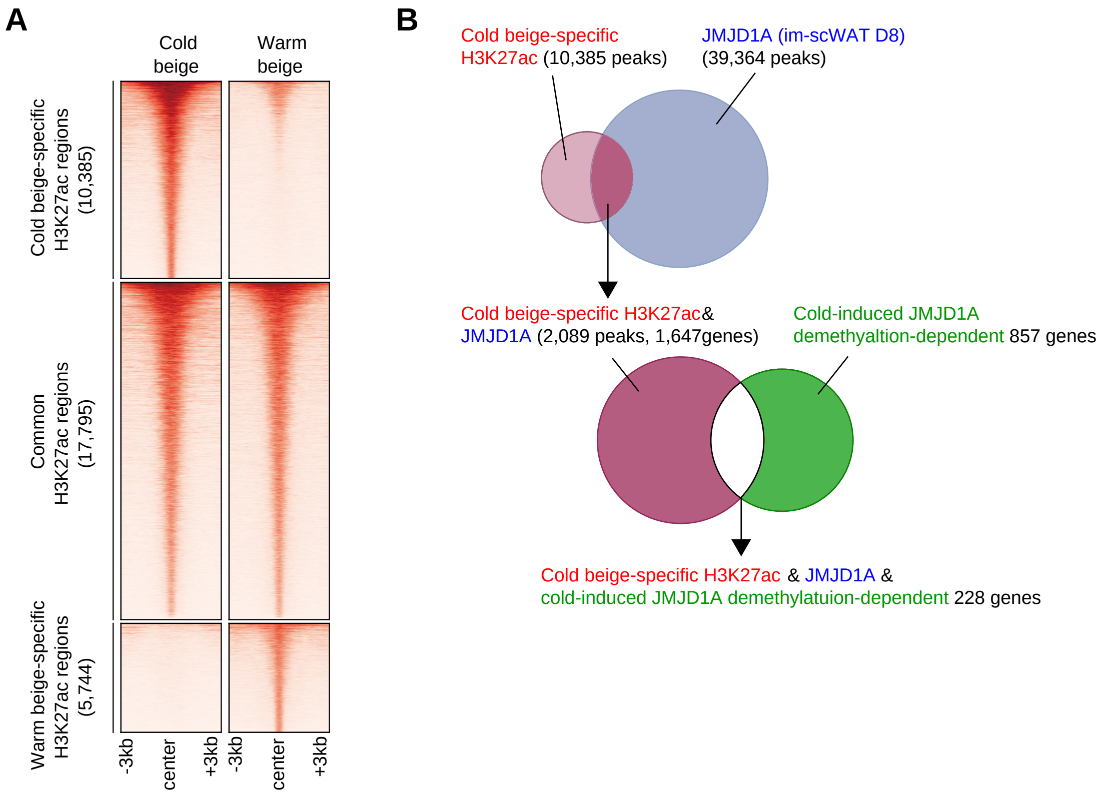

Figure S5. Integration of ChIP-seq and RNA-seq data led to the identification of JMJD1A direct target genes, related to Figure 5.

(A) Classification of H3K27ac regions in cold and warm beige adipocytes isolated from scWAT. Data for H3K27ac ChIP-seq of cold and warm beige adipocytes were obtained from GSE108077[S1].

(B) Venn diagram showing the overlap between JMJD1A binding sites of im-scWAT beige cultures on D8 (39,364 peaks) and cold beige-specific H3K27ac regions (10,385 peaks) (top). Overlapping regions were defined as cold beige-specific H3K27ac and JMJD1A binding regions (2,089 peaks). Venn diagram showing the cold-induced JMJD1A demethylation-dependent, cold beige-specific H3K27ac, and JMJD1A binding genes (bottom).

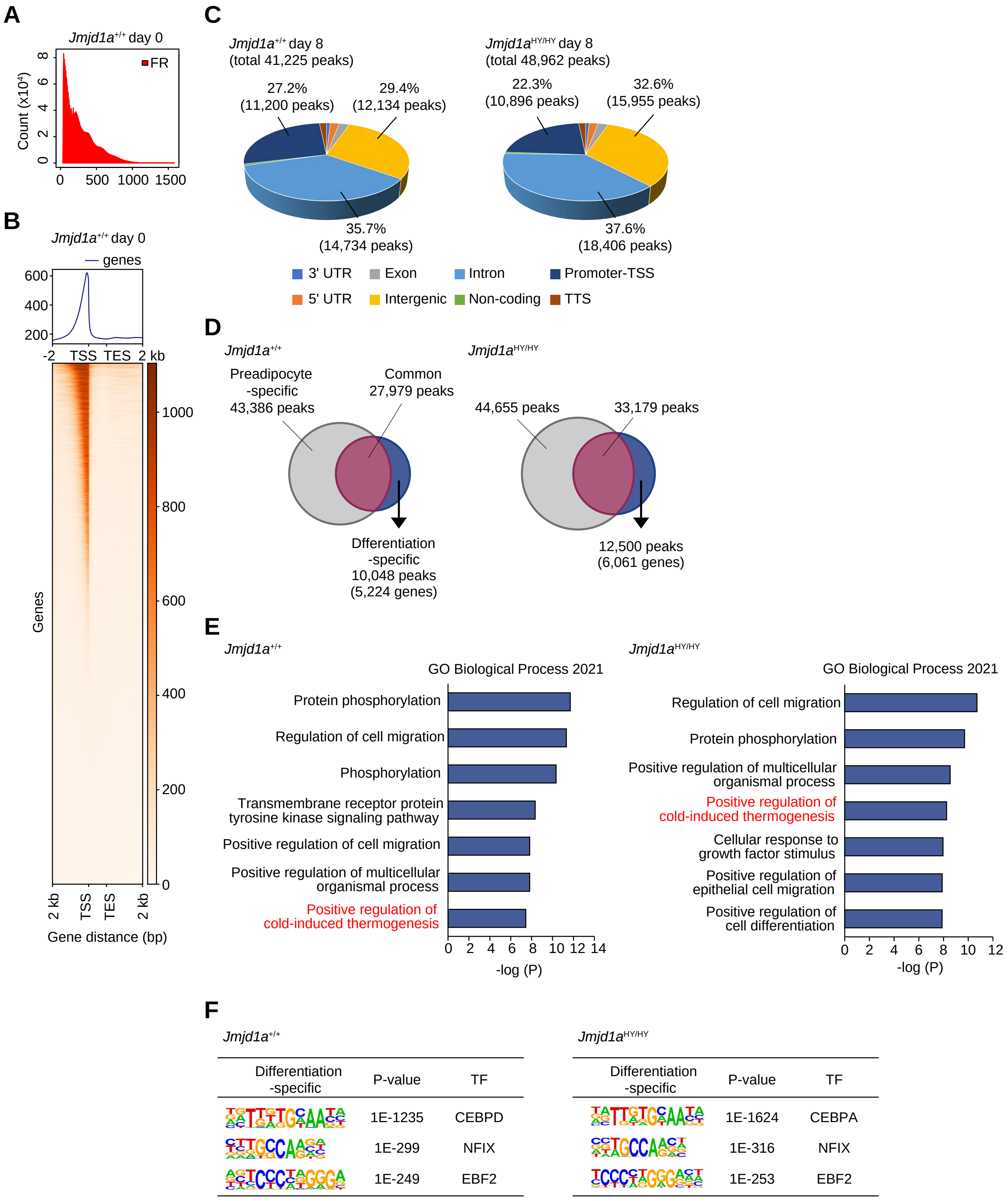
A
Jmjd1a+/+ day 0
FR
Count (x10⁴)
0 2 4 6 8
0 500 1000 1500
B
Jmjd1a+/+ day 0
genes
600
400
200
-2 TSS TES 2 kb
1000
800
600
400
200
0
Genes
2 kb TSS TES 2 kb
Gene distance (bp)
C
Jmjd1a+/+ day 8
(total 41,225 peaks)
27.2%
(11,200 peaks)
29.4%
(12,134 peaks)
35.7%
(14,734 peaks)
Jmjd1aHY/HY day 8
(total 48,962 peaks)
22.3%
(10,896 peaks)
32.6%
(15,955 peaks)
37.6%
(18,406 peaks)
3' UTR
Exon
Intron
Promoter-TSS
5' UTR
Intergenic
Non-coding
TTS
D
Jmjd1a+/+
Preadipocyte
-specific
43,386 peaks
Common
27,979 peaks
Dfferentiation
-specific
10,048 peaks
(5,224 genes)
Jmjd1aHY/HY
44,655 peaks
33,179 peaks
12,500 peaks
(6,061 genes)
E
Jmjd1a+/+
GO Biological Process 2021
Protein phosphorylation
Regulation of cell migration
Phosphorylation
Transmembrane receptor protein tyrosine kinase signaling pathway
Positive regulation of cell migration
Positive regulation of multicellular organismal process
Positive regulation of cold-induced thermogenesis
0 2 4 6 8 10 12 14
-log (P)
Jmjd1aHY/HY
GO Biological Process 2021
Regulation of cell migration
Protein phosphorylation
Positive regulation of multicellular organismal process
Positive regulation of cold-induced thermogenesis
Cellular response to growth factor stimulus
Positive regulation of epithelial cell migration
Positive regulation of cell differentiation
0 2 4 6 8 10 12
-log (P)
F
Jmjd1a+/+
Differentiation -specific
P-value
TF
1E-1235
CEBPD
1E-299
NFIX
1E-249
EBF2
Jmjd1aHY/HY
Differentiation -specific
P-value
TF
1E-1624
CEBPA
1E-316
NFIX
1E-253
EBF2

Figure S6. Validation of ATAC-seq analysis of differentiated beige cells, related to Figure 5.
(A) Fragment size distribution of the ATAC-seq library of im-scWAT from *Jmjd1a*$^{+/+}$ mice on day 0.
(B) Metaplots showing the abundance of ATAC-seq signal intensity in im-scWAT cells from *Jmjd1a*$^{+/+}$ mice on day 0 (top). The heatmap shows the accessibility of all genes (bottom).
(C) Genome-wide distribution of ATAC-seq signals in differentiated im-scWAT derived from *Jmjd1a*$^{+/+}$ or *Jmjd1a*$^{HY/HY}$. TSS, transcription start site. TTS, transcription termination site.
(D) Venn diagram showing preadipocyte (day 0) and beige adipocyte (day 8) -derived peaks of *Jmjd1a*$^{+/+}$ (left) or *Jmjd1a*$^{HY/HY}$ (right) cells The gray, red, and blue regions are defined as preadipocyte-specific regions (43,386 or 44,655 peaks), common regions (27,979 or 33,179 peaks), and differentiation-specific regions (10,048 or 12,500 peaks), respectively.
(E) Gene Ontology (GO) analysis of 5,224 or 6,061 genes annotated from differentiation-specific peaks in im-scWAT cells from *Jmjd1a*$^{+/+}$ (left) and *Jmjd1a*$^{HY/HY}$ (right) mice, respectively. “Positive regulation of cold-induced thermogenesis" was significantly enriched in both groups.
(F) HOMER motif analysis of the differentiation-specific peaks.

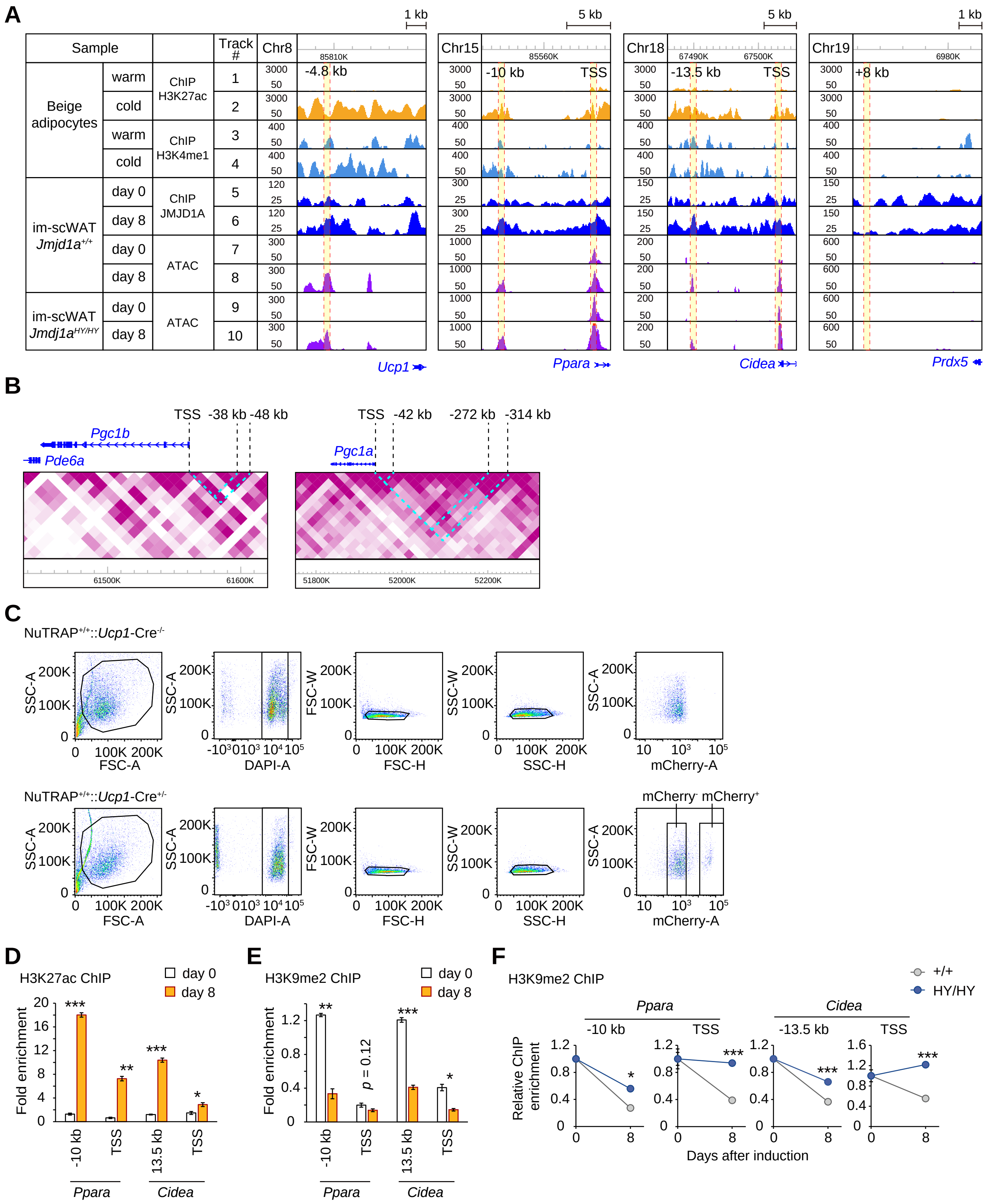

A
Sample
Track #
Beige adipocytes
warm
cold
ChIP H3K27ac
ChIP H3K4me1
im-scWAT Jmjd1a+/+
day 0
day 8
ChIP JMJD1A
ATAC
im-scWAT Jmjd1aHY/HY
Chr8
Chr15
Chr18
Chr19
-4.8 kb
-10 kb
TSS
-13.5 kb
+8 kb
Ucp1
Ppara
Cidea
Prdx5
B
TSS
-38 kb
-48 kb
-42 kb
-272 kb
-314 kb
Pgc1b
Pde6a
Pgc1a
C
NuTRAP+/+::Ucp1-Cre-/-
NuTRAP+/+::Ucp1-Cre+/-
SSC-A
FSC-A
DAPI-A
FSC-W
FSC-H
SSC-W
SSC-H
mCherry-A
mCherry- mCherry+
D
H3K27ac ChIP
day 0
day 8
Fold enrichment
-10 kb
TSS
13.5 kb
Ppara
Cidea
E
H3K9me2 ChIP
p = 0.12
F
+/+
HY/HY
Relative ChIP enrichment
Days after induction

Figure S7. JMJD1A demethylates H3K9me2 in the enhancer region of mitochondrial regulator and thermogenesis genes, related to Figure 5.

(A) Genome browser representation for H3K27ac and H3K4me1 in cold or warm beige adipocytes and JMJD1A and ATAC in im-scWAT on days 0 and 8 in *Ucp1*, *Ppara*, *Cidea* and *Prdx5* genomic regions.

(B) 2D heat map showing the interaction frequency of *Pgc1b* (left) and *Pgc1a* (right) genomic regions in the Hi-C map.

(C) Gating strategy for the purification of nuclei specifically derived from beige adipocytes of NuTRAP$^{+/+}$::*Ucp1*-Cre$^{+/-}$ mice using a flow cytometer. After debris removal using the FSC-A/SSC-A scatter gate, the nuclei were gated using DAPI signals. Doublets were then excluded using FSC-H/FSC-W and SSC-H/SSC-W gates. Target populations were collected based on mCherry fluorescence. NuTRAP$^{+/+}$::*Ucp1*-Cre$^{-/-}$ mice were used as gates for mCherry-positive or -negative nuclei.

(D and E) ChIP-qPCR analysis of H3K27ac (D) and H3K9me2 (E) in the indicated *Ppara* and *Cidea* enhancers and TSS during beige adipogenesis.

(F) Changes in H3K9me2 levels determined by ChIP-qPCR of *Ppara* and *Cidea* during beige adipogenesis in im-scWAT from *Jmjd1a*$^{+/+}$ and *Jmjd1a*$^{HY/HY}$ mice.

Data are expressed as mean ± SEM of three technical replicates (D-F). Representative of two (D, E) or three (F) independent experiments. Welch's t test (D-F) was used for comparisons. *p < 0.05, **p < 0.01 and ***p < 0.001 were considered statistically significant.

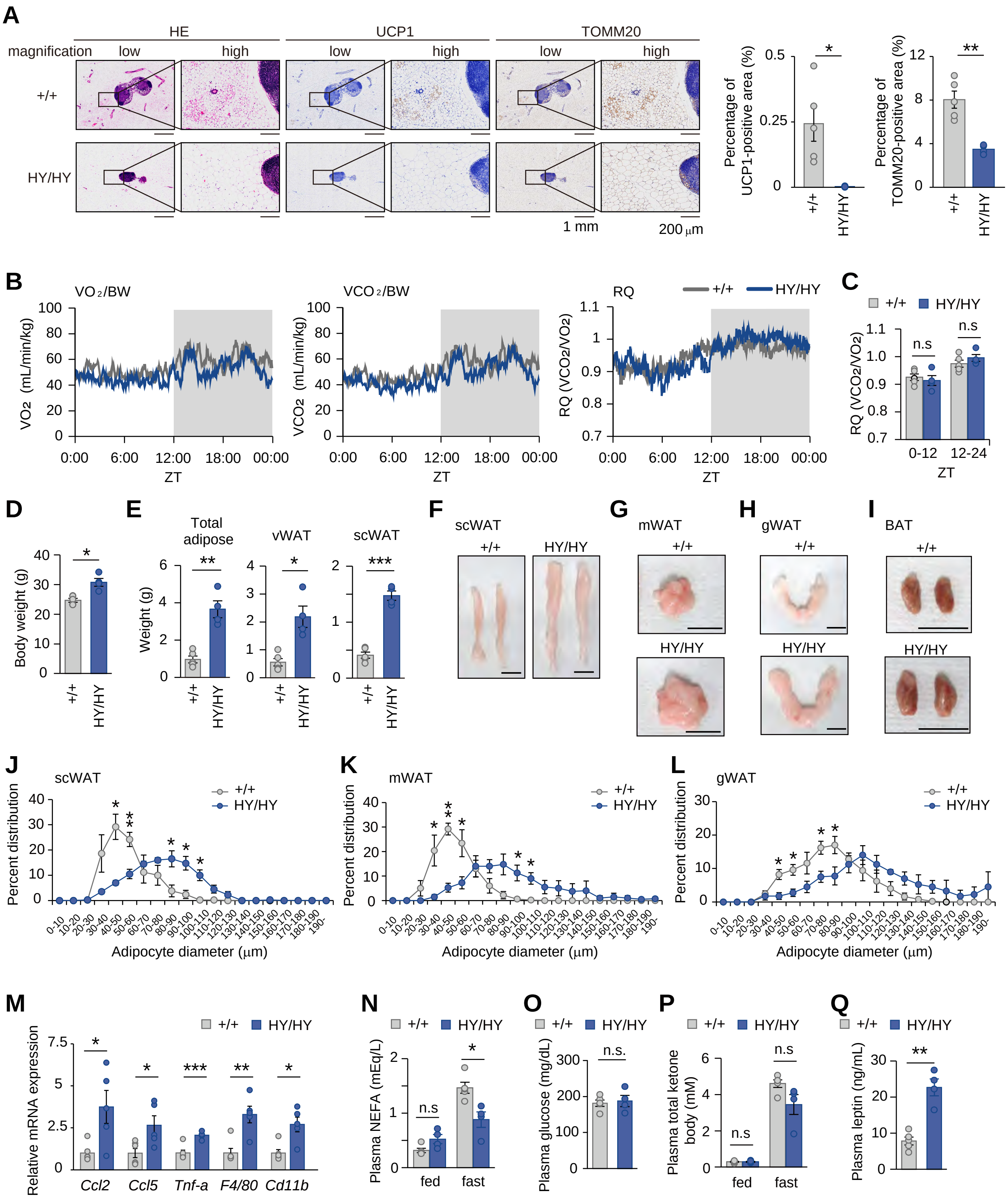
A
HE
UCP1
TOMM20
magnification
low
high
+/+
HY/HY
1 mm
200 μm
Percentage of UCP1-positive area (%)
Percentage of TOMM20-positive area (%)
B
VO2/BW
VCO2/BW
RQ
VO2 (mL/min/kg)
VCO2 (mL/min/kg)
RQ (VCO2/VO2)
ZT
C
n.s
0-12
12-24
D
Body weight (g)
E
Total adipose
vWAT
scWAT
Weight (g)
F
scWAT
G
mWAT
H
gWAT
I
BAT
J
scWAT
K
mWAT
L
gWAT
Percent distribution
Adipocyte diameter (μm)
M
Relative mRNA expression
Ccl2
Ccl5
Tnf-a
F4/80
Cd11b
N
Plasma NEFA (mEq/L)
fed
fast
O
Plasma glucose (mg/dL)
n.s.
P
Plasma total ketone body (mM)
Q
Plasma leptin (ng/mL)

Figure S8. Obesity and metabolic disorders caused by impaired mitochondrial biogenesis, related to Figure 6.

(A) Low-magnification images of H&E, UCP1 and TOMM20 staining in scWAT sections from *Jmjd1a*$^{+/+}$ and *Jmjd1a*$^{HY/HY}$ mice as shown in Fig. 6E (left; scale bar, 1 mm at low magnification or 200 μm at high magnification). Percentage of UCP1- or TOMM20-positive area were calculated per the field of view (right) (*Jmjd1a*$^{+/+}$: n = 5, *Jmjd1a*$^{HY/HY}$: n = 4).

(B) Oxygen consumption (VO_2), carbon dioxide production (VCO_2), and respiratory quotient (RQ) of *Jmjd1a*$^{+/+}$ and *Jmjd1a*$^{HY/HY}$ mice were measured over 24 h. VO_2 and VCO_2 were normalized to the mouse body weight (*Jmjd1a*$^{+/+}$: n = 5, *Jmjd1a*$^{HY/HY}$: n = 4).

(C) Mean RQ calculated every 12 h.

(D) Differences in body weight between *Jmjd1a*$^{+/+}$ and *Jmjd1a*$^{HY/HY}$ mice subjected to CT (Figure 6G).

(E) Quantification of fat mass from CT scans of the total fat, vWAT, and scWAT (*Jmjd1a*$^{+/+}$: n = 5, *Jmjd1a*$^{HY/HY}$: n = 4).

(F-I) Representative images of scWAT (F), mWAT (G), gWAT (H), and BAT (I).

(J-L) Line graph showing the distribution of adipocyte diameters in the scWAT (J), mWAT (K), and gWAT (L) of *Jmjd1a*$^{+/+}$ and *Jmjd1a*$^{HY/HY}$ mice (*Jmjd1a*$^{+/+}$: n = 5, *Jmjd1a*$^{HY/HY}$: n = 4).

(M) mRNA levels of *Ccl2*, *Ccl5*, *Tnf-a*, *F4/80* and *Cd11b* were measured by qPCR of eWAT from *Jmjd1a*$^{+/+}$ or *Jmjd1a*$^{HY/HY}$ mice (*Jmjd1a*$^{+/+}$: n = 6, *Jmjd1a*$^{HY/HY}$: n = 5).

(N and P) Plasma NEFA (N) and total ketone body (P) concentrations were measured in *Jmjd1a*$^{+/+}$ and *Jmjd1a*$^{HY/HY}$ mice that were fed and fasted for 12 h.

(O and Q) Plasma glucose (O) and leptin (Q) levels were measured in *Jmjd1a*$^{+/+}$ and *Jmjd1a*$^{HY/HY}$ mice in fed state.

Data are mean ± SEM (A-E, J-Q). Welch's t test was performed for comparisons (A, C-E, J-Q). **p* < 0.05, ***p* < 0.01 and ****p* < 0.001 were considered statistically significant. n.s indicates no significance.

Figure 1J

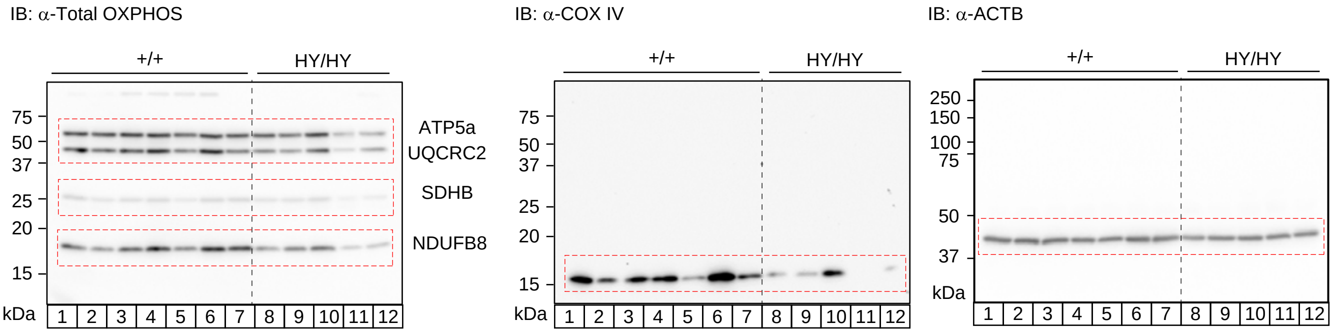

Figure 3F

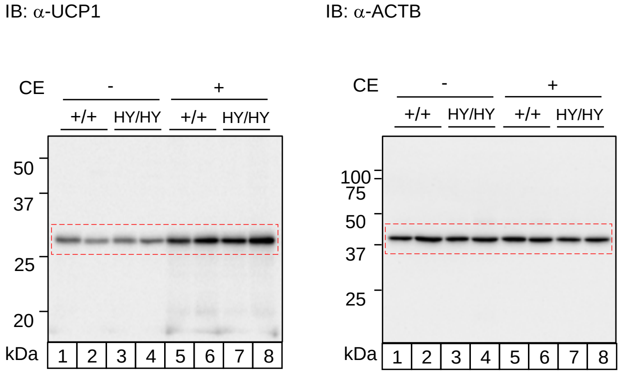

Figure 3G

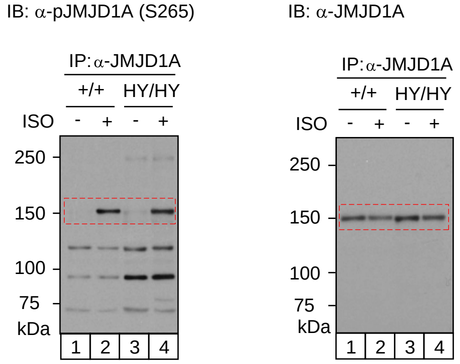

Figure S1H

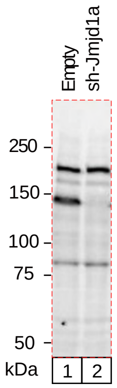

Figure S1I

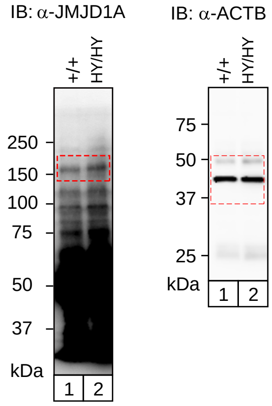

Figure S9. The uncropped images of the blots, related to Figures 1, 3 and S1.

Table S1. List of JMJD1A direct target genes, related to Figure 5.

228 genes which exhibited cold beige-specific H3K27ac and JMJD1A recruitment, and were induced by chronic cold exposure (> 1.5 in scWAT of *Jmjd1a*$^{+/+}$ mice) and reduced in *Jmjd1a*$^{HY/HY}$ mice (< 0.67 at 8ºC).

1190007I07Rik	*Chchd3*	*E2f2*	*Higd1a*	*Mtfp1*	*Pgam1*	*Slc52a2*
2300009A05Rik	*Cidea*	*Eci1*	*Hpdl*	*Mtor*	*Pgls*	*Sod2*
4930415O20Rik	*Clstn3*	*Edf1*	*Idh3a*	*Myl3*	*Phospho1*	*Spc24*
6430571L13Rik	*Coa3*	*Elovl3*	*Impa2*	*Ncapd2*	*Pim1*	*Sucla2*
Aars2	*Coa5*	*Eno1*	*Isca1*	*Ndufa9*	*Pimreg*	*Suclg1*
Abcb8	*Col27a1*	*Etfdh*	*Isca2*	*Ndufab1*	*Pkn1*	*Tbkbp1*
Abcd3	*Coq10a*	*F2r*	*Itpk1*	*Ndufaf4*	*Plbd1*	*Tectb*
Acadl	*Coq8a*	*Fabp3*	*Itpr2*	*Ndufb10*	*Plin2*	*Tfrc*
Acadm	*Coq9*	*Fam210a*	*Khdrbs3*	*Ndufb5*	*Plin5*	*Timm44*
Acadvl	*Cox10*	*Fam57b*	*Letm1*	*Ndufb6*	*Pop5*	*Tmem14c*
Acot12	*Cox5a*	*Fastk*	*Letmd1*	*Ndufb7*	*Ppara*	*Tmem38b*
Acsf3	*Cox5b*	*Ffar4*	*Lipa*	*Ndufb8*	*Pgc1a*	*Tmem82*
Acsl5	*Cox6a1*	*Fh1*	*Lncbate10*	*Ndufb9*	*Pgc1b*	*Tnfrsf9*
Adcy10	*Cox6c*	*Fndc5*	*Lynx1*	*Ndufs2*	*Ppdpf*	*Traf4*
Adtrp	*Cox7a2*	*Fuom*	*Lyrm7*	*Ndufs4*	*Prelid3b*	*Tspan18*
Afg1l	*Cox7c*	*Fxn*	*Mcrip2*	*Ndufs6*	*Pyurf*	*Ttc25*
Ahcyl1	*Cox8a*	*Gapdh*	*Mecr*	*Ndufs7*	*Qrsl1*	*Tufm*
Ak1	*Cox8b*	*Gja1*	*Micos10*	*Ndufs8*	*Rilp*	*Txn2*
Apln	*Cpne5*	*Gk*	*Micos13*	*Ndufv1*	*Rmdn1*	*Tysnd1*
Aspg	*Crls1*	*Gm11827*	*Mpc1*	*Ndufv2*	*Rnf152*	*Ube2t*
Aste1	*Cs*	*Gm14057*	*Mpc2*	*Ndufv3*	*S1pr5*	*Ucp1*
Atf5	*Cyc1*	*Gm15179*	*Mrpl12*	*Nectin2*	*Samm50*	*Ucp3*
Atox1	*Cyp2u1*	*Gm32200*	*Mrpl15*	*Nek2*	*Sdhb*	*Uqcr10*
Atp5b	*Dagla*	*Gm9899*	*Mrpl34*	*Nrp2*	*Sdr39u1*	*Uqcr11*
Atp5j2	*Ddhd2*	*Gmnn*	*Mrpl38*	*Ogdh*	*Shb*	*Uqcrb*
Atp5l	*Ddo*	*Got1*	*Mrpl4*	*Oplah*	*Slc16a1*	*Uqcrc2*
Atp5md	*Decr1*	*Gpd2*	*Mrpl42*	*Pank1*	*Slc25a19*	*Uqcrfs1*
Atp5o	*Deptor*	*Grk3*	*Mrps23*	*Pde4a*	*Slc25a20*	*Uqcrq*
B330016D10Rik	*Dhrs7*	*Grtp1*	*Mrps27*	*Pdhb*	*Slc25a34*	*Vwa8*
Btbd1	*Dhrs9*	*Gtf2ird1*	*Mrps33*	*Pdk2*	*Slc25a39*	*Zfp691*
Ccdc58	*Dlst*	*Hadh*	*Mrps35*	*Pdk4*	*Slc25a42*	
Ccr10	*Dnaja3*	*Hadha*	*Mrps36*	*Pebp1*	*Slc25a51*	
Cdca2	*Dnajc11*	*Hadhb*	*Mtch2*	*Pfkl*	*Slc4a4*	

Table S2. Details of the sex and age of mice, related to STAR Methods.

Mouse strain	Sex	Age (at starting point of the treatment)	Figure
C57BL/6J	Male	12	Figure 1B, Figure S1A
Jmjd1a$^{+/+}$ and *Jmjd1a*$^{HY/HY}$ mice	Female	25	Figure 1H, J
	Female	16	Figure 1I, 2B, C, 4B-H, Figure S2A-F, 3A-G
	Female	14	Figure 2D,
	Female	18	Figure S2G
	Female	13	Figure 3B
	Female	22	Figure 3C-F
NuTRAP$^{+/+}$::*Ucp1*-Cre$^{+/-}$	Male	29	Figure 5B-D, Figure S6C
Jmjd1a$^{+/+}$ and *Jmjd1a*$^{HY/HY}$ mice	Male	2	Figure 6A
	Female	5	Figure 6B
	Female	53	Figure 6C-E, H, I, Figure S8A, F-M
	Male	57	Figure 6H (eWAT), I (eWAT)
	Female	42	Figure 6F, Figure S8B, C
	Female	43	Figure 6G, Figure S8D, E
	Female	50	Figure 6J-L, Figure S8N-P
	Female	49	Figure 6M, N
	Female	52	Figure 6O
	Female	8	Figure 6P-S
	Female	15	Figure 6T

Table S3. List of RT-qPCR primers, related to STAR Methods.

Gene	Sequence	
	Forward Primer	Reverse Primer
Cyclophilin	5’-GGAGATGGCACAGGAGGAA-3’	5’-GCCCGTAGTGCTTCAGCTT-3’
Rpl32	5’-GGACCAAGAAGTTCATCAGGC-3’	5’-TCCCATAACCGATGTTGGGC-3’
Pparg	5’-CAAGAATACCAAAGTGCGATCAA-3’	5’-GAGCTGGGTCTTTTCAGAATAATAAG-3’
Ucp1	5’-AAGCTGTGCGATGTCCATGT-3’	5’-AAGCCACAAACCCTTTGAAAA-3’
Cidea	5’-GGTTCAAGGCCGTGTTAAGG-3’	5’-CGTCATCTGTGCAGCATAGG-3’
Elovl3	5’-TTCTCACGCGGGTTAAAAATG-3’	5’-GGGCCTTAAGTCCTGAAACGT-3’
Dio2	5’-CTTCCTCCTAGATGCCTACAAAC-3’	5’-GGCATAATTGTTACCTGATTCAGG-3’
Pgc1a	5’-AACCACACCCACAGGATCAGA-3’	5’-TCTTCGCTTTATTGCTCCATGA-3’
Pgc1b	5’-GAGGGCTCCGGCACTTC-3’	5’-CGTACTTGCTTTTCCCAGATGA-3’
Ndufs8	5’-GGAGAGGAGCGTTGCATTG-3’	5’-ATGTCATAGCGTGTCGTTCGG-3’
Cox5a	5’-ATGCCTGGGAATTGCGTAAAG-3’	5’-TGCGAACAGCACTAGCAAAAT-3’
Atp5j2	5’-TGCCGAGCTGGATAATGATGC-3’	5’-ACCATGCTAATCCCCGAGATG-3’
Sdhb	5’-AATTTGCCATTTACCGATGGGA-3’	5’-AGCATCCAACACCATAGGTCC-3’
mt-Nd1	5'-GTTGGTCCATACGGCATTTT-3'	5'-GTTGGTCCATACGGCATTTT-3'
mt-Nd2	5'-GCCTGGAATTCAGCCTACTAGC-3'	5'-GGCTGTTGCTTGTGTGACGA-3'
mt-Nd3	5'-TCCGAGCATCTTATCCACGC-3'	5'-GTATGGTGGTACTCCCGCTG-3'
mt-Nd4	5'-CGCCTACTCCTCAGTTAGCCA-3'	5'-TGATGTGAGGCCATGTGCGA-3'
Ccl2	5'-CTTCTGGGCCTGCTGTTCA-3'	5'-CCAGCCTACTCATTGGGATCA-3'
Ccl5	5'-TGCCCTCACCATCATCCTCACT-3'	5'-GGCGGTTCCTTCGAGTGACA-3'
F4/80	5'-CTTTGGCTATGGGCTTCCAGTCC-3'	5'-GCAAGGAGGACAGAGTTTATCGTG-3'
Cd11b	5'-AACTTCACGGCTTCAGAGATG-3'	5'-AGGCACTTGAGAGGTTCTGG-3'
Tnf-a	5'-CATCTTCTCAAAATTCGAGTGACAA-3'	5'-CCAGCTGCTCCTCCACTTG-3'

Table S4. List of ChIP-qPCR primers, related to STAR Methods.

Amplified regions	Sequence	
	Forward Primer	Reverse Primer
Pgc1b -48 kb	5'-ACCATACACCAGCCATGTGA-3'	5'-ACCACCCTCCAGAGACCTTT-3'
Pgc1b -38 kb	5'-GACCCTTGTTGGTCCTTTGA-3'	5'-GGGCACAGTTACAGGCAAGT-3'
Pgc1a -314 kb	5'-TGCCACCACACAACCTTTTA-3'	5'-CCAAATTGGGCAAAGGAGTA-3'
Pgc1a -272 kb	5'-TCTGCAAGGTTGCCAAATAA-3'	5'-AAGGGCTCTCAAAGGTCCAT-3'
Pgc1a -42 kb	5'-TTCCCTGTGTTTTCCACTCC-3'	5'-GATCCTTGGCTTAGCCTGTG-3'
Ucp1 -4.8 kb	5'-TGCAACCCCTCACCTTTTAC-3'	5'-CTCCTTCCATCATCCCTTCA-3'
Prdx5 +8.0 kb	5'-ACAAGCAGCACTGAACAGAC-3'	5'-GAGTTGCCTTGGTCATGGTG-3'
Ppara -10 kb	5'-TGGCCGGGAGGAACTG-3'	5'-GGCAGGGACAATCTCTTTGTG-3'
Ppara TSS	5'-GGCAGTCCCTTCACCTAACC-3'	5'-TCCTCGATGCCCATTTAGTG-3'
Cidea -13.5 kb	5'-CACCGCTTCACTTTGTCCTTT-3'	5'-GAGCACCCGGTTTGACAGT-3'
Cidea TSS	5'-CACGCACACCTGCTTCTCTA-3'	5'-GATGTTGGTGGCTCTTGTCA-3'

SUPPLEMENTAL REFERENCE

S1. Roh, H.C., Tsai, L.T.Y., Shao, M., Tenen, D., Shen, Y., Kumari, M., Lyubetskaya, A., Jacobs, C., Dawes, B., Gupta, R.K., and Rosen, E.D. (2018). Warming Induces Significant Reprogramming of Beige, but Not Brown, Adipocyte Cellular Identity. Cell Metab 27, 1121-1137 e1125. 10.1016/j.cmet.2018.03.005.
